# A Permeable Triboelectric Fiber Mat with 35 V cm^−2^ Voltage Output for Wearable Wireless Sensing Electronics

**DOI:** 10.1002/smll.202504556

**Published:** 2025-07-02

**Authors:** Youchao Qi, Jinxing Jiang, Fan Chen, Junhua Zhou, Jiaheng Liang, Jingjing Fu, Yongqiang Yang, Yichun Ding, Zijian Zheng, Qiyao Huang

**Affiliations:** ^1^ School of Fashion and Textiles The Hong Kong Polytechnic University Hong Kong SAR 999077 P. R. China; ^2^ Department of Applied Biology and Chemical Technology Faculty of Science The Hong Kong Polytechnic University Hong Kong SAR 999077 P. R. China; ^3^ Research Institute for Intelligent Wearable Systems The Hong Kong Polytechnic University Hong Kong SAR 999077 P. R. China; ^4^ Research Institute for Smart Energy The Hong Kong Polytechnic University Hong Kong SAR 999077 P. R. China; ^5^ PolyU‐Wenzhou Technology and Innovation Research Institute Wenzhou Zhejiang 325024 P. R. China

**Keywords:** electrospinning, liquid metal, permeable triboelectric fiber mat, self‐powered system, wireless temperature monitoring

## Abstract

Textile‐based triboelectric nanogenerators have emerged as a promising solution for self‐powered wearable electronics, owing to their exceptional comfort derived from the inherent flexibility of textiles, coupled with their remarkable capability to efficiently harvest low‐frequency energy from human motions. However, one primary challenge lies in how to enhance output and management efficiency without compromising comfort to meet the high‐power consumption demands of electronics. Herein, a permeable triboelectric nanogenerator (*p*TENG) is reported with a voltage output exceeding 35 V cm^−^^2^ while maintaining breathability. Such a high output of this *p*TENG is attributed to the enhanced dielectric constant, facilitated by the uniform distribution of liquid metal nanoparticles in the electrospun composite fiber mat. With a specially designed energy management module, the self‐powering system based on *p*TENG can achieve 10 times faster charging speed than those regulated only by rectifiers. As a proof‐of‐concept demonstration, a garment integrating a *p*TENG, an energy management module, a temperature sensor, and a wireless transmitter is developed to form a self‐powered wireless temperature sensing system, which can sense and transmit temperature data to a relay terminal module. This integration reduces reliance on external power while enabling real‐time wireless health monitoring, highlighting the great potential of body area networks in personalized healthcare.

## Graphical abstract

This work presents a textile‐based permeable triboelectric nanogenerator (*p*TENG) with a remarkable output of 35 V cm^−^^2^, enabled by an electrospun composite fiber mat embedded with liquid metal nanoparticles. Integrated with a custom‐designed energy management module, it powers a wireless temperature sensing system, showcasing strong potential for self‐powered wearable electronics and body area networks in personalized healthcare and real‐time physiological monitoring.

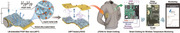

## 1 Introduction

Energy harvesting technology can capture ambient energies, such as mechanical, solar, and thermal energy, and convert them into electrical power.^[^ ^1^, ^2^, ^3^, ^4^, ^5^ ^]^ This technology is especially appealing to wearable wireless sensing electronics used for sports and rehabilitation exercises, where activities such as running, walking, and joint movements can be harnessed for the powering of wireless sensor electronics without the need for batteries. As a result, an uninterrupted energy supply during usage can be offered, leading to lower maintenance costs, longer device lifespan, and greater environmental friendliness compared to battery‐powered systems. Commonly utilized energy harvesting technologies for extracting mechanical energy from the human body include electromagnetic induction,^[^ ^6^, ^7^, ^8^ ^]^ piezoelectric,^[^ ^9^, ^10^, ^11^, ^12^ ^]^ and electrostatic generators.^[^ ^13^, ^14^ ^]^ They, however, struggle to efficiently capture the energy from the low‐frequency mechanical movements of the human body (typically < 10 Hz) due to their limited conversion capabilities to lower frequency motions (generally output voltage <0.2 V).^[^ ^15^, ^16^ ^]^ Triboelectric nanogenerators (TENGs),^[^ ^17^, ^18^, ^19^, ^20^ ^]^ an energy‐harvesting technology that is based on the coupling effects of contact electrification and electrostatic induction, has been proposed as an alternative. They exhibit an inherently high voltage output (>10−100 V) that is independent of frequency, allowing most of the generated electrical energy to sufficiently power electronic devices operate at a threshold voltage of ≈0.2−4 V.^[^ ^16^ ^]^ In addition, TENGs offer advantages such as wide selection of materials,^[^ ^21^, ^22^, ^23^, ^24^ ^]^ lightweight design,^[^ ^25^, ^26^ ^]^ low cost,^[^ ^27^ ^]^ and ease of scalability through advanced structural engineering.^[^ ^28^ ^]^ Such characteristics enable the TENGs to be more adaptable at harvesting micromechanical energy generated by human motion compared to counterparts.

To date, TENGs have attracted widespread research interest for powering smart wireless sensing electronics.^[^ ^29^, ^30^, ^31^, ^32^ ^]^ The state‐of‐the‐art TENGs can achieve high voltage output typically exceeding 30 V cm^−2^, which can meet the energy requirement of most wearable wireless sensing electronics. TENGs with high voltage output are primarily developed by using highly electronegative thin‐film materials (such as polytetrafluoroethylene, fluorinated ethylene propylene, etc.).^[^ ^33^ ^]^ However, the lack of permeability to air and moisture in these thin‐film materials poses a barrier to practical applications in prolonged use, potentially leading to wearing discomfort over extended periods.^[^ ^34^ ^]^ Developing TENGs on the basis of textile materials and structures shows promise in addressing the lack of permeability in thin‐film devices, which is attributed to the fibrous configuration of textiles that can impart intrinsic breathability to TENG textiles.^[^ ^35^ ^]^ As such, textile‐based TENGs can not only offer excellent softness, air and moisture permeability that leads to wearing comfort characteristic, but also the ability to generate electricity from the body movement to power wearable sensors. Nevertheless, textile‐based TENGs encounter hurdles in meeting the power demands of wireless sensing electronics that typically possess high power consumption requirements (µW ∼ mW).^[^ ^36^ ^]^ This is due to the relatively low output voltage (typically <30 V cm^−^^2^) of textile‐based TENGs compared to those of the thin‐film TENGs, and the inherent limitation of pulsed alternating voltage in powering electronics that require a stable direct voltage of 2.5–4 V. Although energy management technology, such as rectification and buck‐boost conversion, has been developed to transform the pulsed alternating current output of TENGs into a stable direct current output for reliably powering the sensors,^[^ ^37^, ^38^ ^]^ challenging still persist in boosting the output of TENG textiles and maximizing the efficient utilization of the generated energy.

To address the above‐mentioned challenge, we herein report a permeable triboelectric nanogenerator (*p*TENG) with a high output voltage of over 35 V cm^−2^. *p*TENG is composed of a high‐performance triboelectric composite fiber mat (denoted as LMPT), which is developed by the electrospinning of liquid‐metal (LM)‐embedded PVDF‐TrFE (Poly(vinylidene fluoride‐co‐trifluoroethylene)). The fibrous structure of the electrospun LMPT composite fiber mat facilitates rapid pathways for the permeation of air and moisture, thereby endowing the *p*TENG with high air permeability (up to 8.8 mm s^−1^) and moisture permeability (up to 630 g m^−^^2^ day^−1^). Importantly, the incorporation of LM nanoparticles in LMPT significantly boosts the output performance of the as‐formed *p*TENG, due to the increased dielectric constant of the LMPT. *p*TENG consisting of LMPT possesses a exceptionally high voltage output exceeding 35 V cm^−2^ and a high charge density of 3.36 nC cm^−2^, surpassing those of its counterpart (*p*TENG made of PVDF‐TrFE fiber mat without embedding LM nanoparticle) by 83% and 46%, respectively. To maximize the utilization of the generated electricity from *p*TENG, an energy management module incorporating buck‐boost current regulation, active voltage monitoring, and stabilized output control is further designed. By integrating this energy management module, LMPT‐based *p*TENG can charge a commercial 0.68 mF capacitor to 3.1 V in 300 s, showcasing a remarkable ten‐fold enhancement in charging speed compared to the counterpart. As a proof‐of‐concept demonstration, *p*TENGs are utilized to rapidly power a wireless sensing system with power consumption of ≈22.5 mW, facilitating the sensing and real‐time transmission of temperature data to the cloud through Wireless Fidelity (Wi‐Fi). This reported work demonstrates the successful integration of human motion energy harvesting, efficient energy management, and wireless sensing technologies. Such an integration enables the real‐time healthcare monitoring while maintaining the wearability of wearable technologies, presenting substantial application potential in areas such as wireless sensing electronics and body area network.

## 2 Results and Discussion

### 2.1 Fabrication and Characterization of LMPT

**Figure 1:**
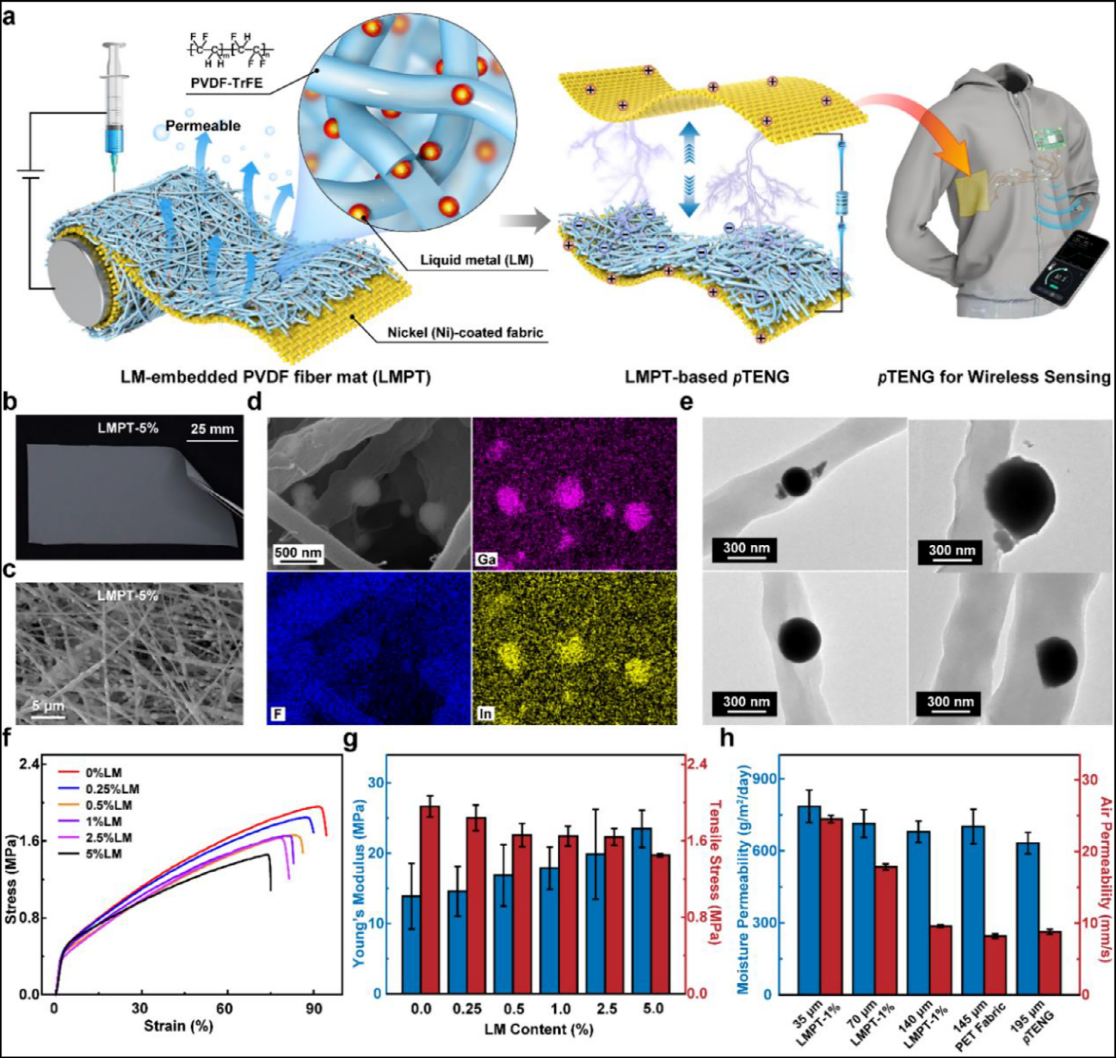
Fabrication and characterization of Liquid‐metal (LM)‐embedded PVDF‐TrFE triboelectric composite fiber mat (denoted as LMPT) for permeable triboelectric nanogenerator (*p*TENG). a) Design of LMPT and its *p*TENG for application in powering wireless sensing. b) Digital image and c) Scanning electron microscopy (SEM) image of LMPT‐5% (5% is the weight ratio of liquid metal to the electrospinning dope). d) SEM elemental mapping of LMPT‐1% showing the distribution of LM particles in the fiber mat (1% is the weight ratio of liquid metal to the electrospinning dope). e) Transmission electron microscopy (TEM) image showing the distribution of LM nanoparticles within the PVDF‐TrFE fibers. f) Stress‐strain curves for the LMPT with varying LM contents. g) Young's modulus and tensile strength of LMPT with varying LM contents (the data were from the mean of five sets of experimental data, and the error bar was taken from the standard deviation). h) Moisture and air permeability of LMPT‐1% with varying thicknesses, *p*TENG, and PET fabric (the data were from the mean of five sets of experimental data, and the error bar was taken from the standard deviation).

The key component for constructing the *p*TENG is LMPT, which is an electrospun triboelectric fiber mat of PVDF‐TrFE embedded with LM nanoparticles (**Figure** **1**a). The fabrication process of the LMPT is described in detail in the Experimental Section. Briefly, the electrospinning dope for LMPT is prepared by mixing a PVDF‐TrFE solution with a stable suspension of LM nanoparticles (Eutectic Gallium (Ga)‐Indium (In) Alloy) at different ratios (Figure S1, Supporting Information). The LM nanoparticle suspension used herein is prepared by ultrasonication, through which the LM is converted into nanosized spheres with diameters approximately ranging from 40 to 450 nm (Figure S2, Supporting Information). LMPT is then fabricated by electrospinning the LM‐mixed PVDF‐TrFE dope onto a piece of nickel (Ni)‐coated fabric (denoted as Ni fabric), resulting in the formation of a single triboelectric electrode. By pairing with another piece of Ni fabric as the opposing triboelectric electrode, an all‐textile‐based *p*TENG is constructed, which can serve as a power source unit for driving wearable wireless electronics.

The introduction of LM significantly influences the morphological and mechanical properties of LMPT. With the inclusion of LM nanoparticles, LMPT exhibits a grey color distinct from the electrospun PVDF‐TrFE (denoted as PVDF) fiber mat without LM loading (Figure 1b; Figure S3, Supporting Information). Scanning electron microscope (SEM) images of LMPT reveal that electrospun fibers, approximately ranging in diameter from 300 nm to 1.3 µm, are randomly arranged within the matrix, with an even distribution of LM beads partially embedded into the fibers (Figure 1c,d). The distribution of LM in the PVDF fibrous matrix is further investigated by Transmission Electron Microscopy (TEM). In addition to being partially embedded into the PVDF fiber due to their oversize compared to the fiber (Figure 1d), LM nanoparticles with dimensions smaller than 300 nm are fully incorporated within the fiber (Figure 1e). It is also noticed that there are large spindles, typically ranging from 2.5 to 5 µm in dimensions, presented in LMPT, which may be attributed to the aggregation of LM nanoparticles combined with the polymeric matrix (Figure S4, Supporting Information). Such a phenomenon becomes more pronounced as the LM content increases from 0.25% to 5% (by weight ratio to the electrospinning dope) (Figure S5, Supporting Information). As a result of these morphological changes, the tensile properties of LMPTs exhibit corresponding variations. In comparison to the PVDF fiber mat without LM loading, all the LMPT specimens display a decrease in the tensile breaking strength.^[^ ^39^ ^]^ The Young's modulus of LMPT shows an opposite trend to its tensile strength, exhibiting enhanced deformation resistance with increasing LM content (Figure 1f,g). While the breaking strain of the PVDF fiber mat can reach up to 92% at a breaking stress of 1.96 MPa, the breaking strain of LMPT with only 0.25% LM loading decreased to 88% with a breaking stress of 1.84 MPa. With a higher LM loading content of 5%, LMPT achieves a strain of only ≈75% under a breaking stress of 1.5 MPa (Figure 1f). The increased LM nanoparticles within the composite fiber mat may lead to the disruption of the polymeric structure, resulting in the decline in mechanical performance.^[^ ^39^, ^40^ ^]^ Nevertheless, the mechanical strength and softness of LMPT well align with those of textile materials and human skin,^[^ ^41^ ^]^ thereby rendering LMPT suitable for applications in wearables and smart textiles. Additionally, the porous nature of the fibrous composite matrix allows LMPTs of various thickness to exhibit excellent air (up to 24 mm s^−1^) and moisture permeability (up to 780 g m^−^^2^ day^−1^) (Figure 1h). When pairing the 35 µm‐LMPT with Ni‐fabrics for the assembly of *p*TENG, such *p*TENG, with a total thickness of 195 µm, still exhibits promising air (8.8 mm s^−1^) and moisture permeability (630 g m^−^^2^ day^−1^). These characteristics, akin to those of conventional textile fabrics, are particularly advantageous for *p*TENGs in applications involving extended collection of kinetic energy over the long term.

### 2.2 Enhanced Electric Output of *p*TENG Enabled by LMPT

**Figure 2:**
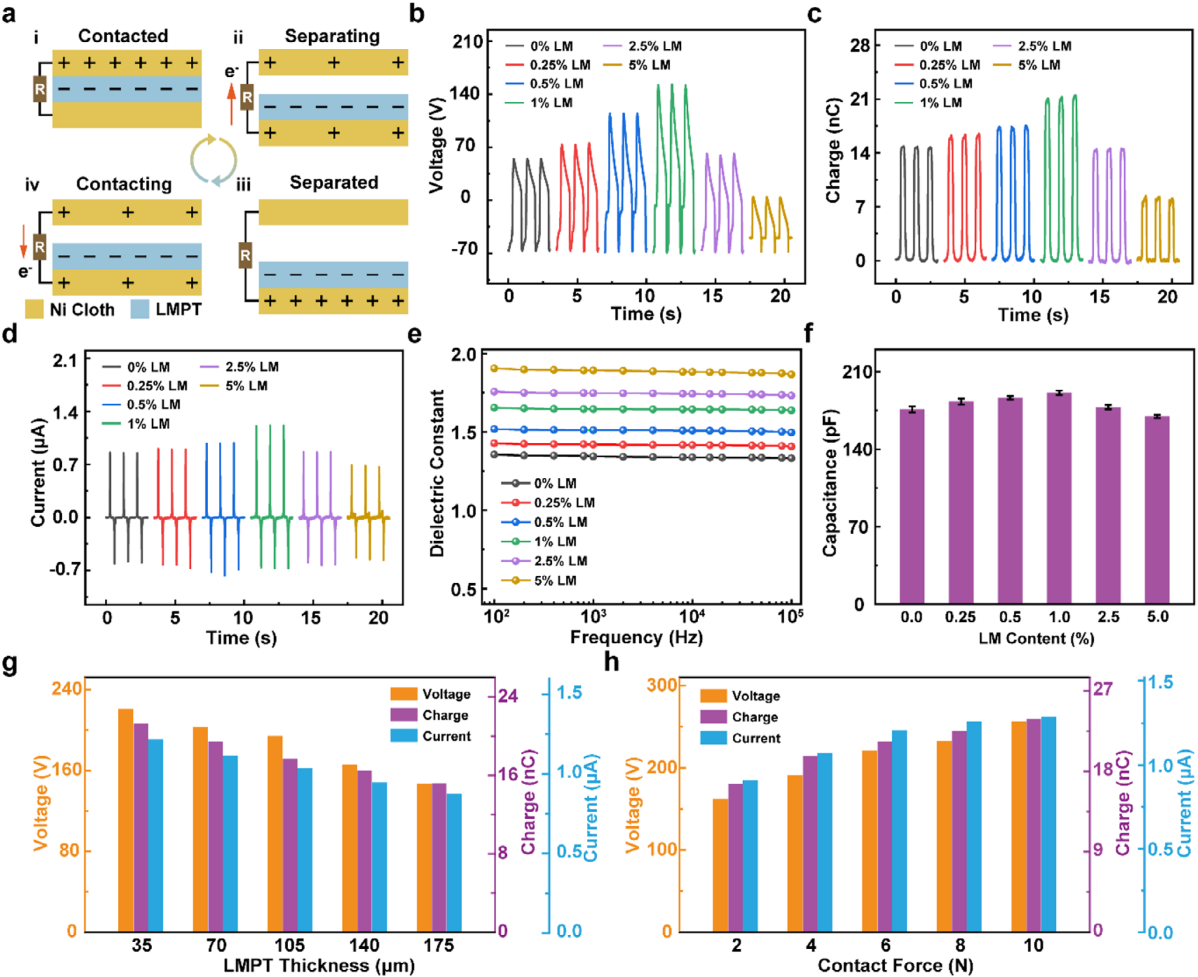
Enhanced electric output of *p*TENG enabled by LMPT. a) Mechanism of electricity generation in *p*TENG. b) Open‐circuit voltage, c) Transferred charge, and d) Short‐circuit current of the *p*TENG assembled with LMPTs of different LM contents. e) Dielectric constant of LMPT with different LM contents. f) Capacitance of the *p*TENG assembled with LMPTs of different LM contents (the data were from the mean of five sets of experimental data, and the error bar was taken from the standard deviation). g,h) Open‐circuit voltage, transferred charge, and short‐circuit current of the *p*TENG at varying LMPT thicknesses and different contact forces.

*p*TENG, consisting of one layer of LMPT spun on Ni fabric as the bottom electrode (negative triboelectric layer) and one layer of Ni fabric as the top electrode (positive triboelectric layer), is assembled for electric output testing. When the two electrodes come into contact, negative charges generated in the bottom electrode transfer from the Ni fabric to the surface of the LMPT, resulting in an equal amount of net charge appearing on the surfaces of the Ni fabric and LMPT, respectively (**Figure** **2**a‐i). When the two pieces of textile start to separate from each other, electrons flow through the external load from the bottom electrode to the top electrode to balance the charges (Figure 2a‐ii). Once the two pieces of textile are fully separated, all electrons are transferred to the top electrode (Figure 2a‐iii). As the two electrodes begin to come into contact again, the electrons on the top electrode flow back to the bottom electrode until full contact is achieved (Figure 2a‐iv). This continuous contact‐separation cycle between the two electrodes generates alternating electrical output. Figure 2b–d exhibits the electric output performance of *p*TENG fabricated with LMPTs of different LM contents. With an increase in LM content to 1%, *p*TENG achieve the voltage, charge, and current output of up to 220 V (≈35 V cm^−2^), 21 nC, and 1.2 µA, respectively, showcasing the performance enhancements of ≈83%, 46%, and 42% compared to the *p*TENG made with pristine PVDF fiber mat without loading LM. To the best of our knowledge, this LMPT‐based *p*TENG represents the highest normalized electric output among the literature of textile‐based TENGs (Table S1, Supporting Information).^[^ ^42^, ^43^, ^44^, ^45^, ^46^, ^47^, ^48^, ^49^, ^50^, ^51^, ^52^, ^53^, ^54^, ^55^, ^56^, ^57^ ^]^ However, further increasing the LM content in the fiber mat composite does not lead to the significant improvement in the electric output of *p*TENG. Instead, the output performance of the LMPT‐based *p*TENG with a 5% loading of LM is even lower than that of the *p*TENG made with pristine PVDF fiber mat.

The enhancement of *p*TENG performance with the increasing loading of LM up to 1% may be attributed to the incorporation of the conductive LM nanoparticles into PVDF fibrous matrix, leading to the trapping of triboelectric charges^[^ ^42^ ^]^ and the increased dielectric constant. During the preparation of LM nanoparticles for electrospinning dope via ultrasonication, the surface of LM rapidly oxidizes to form a Ga_2_O_3_ layer.^[^ ^58^ ^]^ This Ga_2_O_3_ layer, a semiconductor with abundant interface states, plays a crucial role in the transfer process of the triboelectric charges.^[^ ^42^ ^]^ During the contact‐separation cycles of the two triboelectric layers in the *p*TENG (Figure 2a), the accumulation of opposite triboelectric charges leads to the changes in the interface states of Ga_2_O_3_ (Figure S6, Supporting Information). When the LMPT layer is uncharged, Ga_2_O_3_ remains in a flat‐band state, with no electron capture occurring (Figure S6a, Supporting Information). As negative charges accumulate on the LMPT surface, a potential difference arises due to the charge imbalance between the PVDF‐TrFE layer on the LMPT surface and the underlying PVDF‐TrFE fibers. This potential difference drives electrons from the external circuit into the Ni electrode and subsequently into the PVDF‐TrFE fibers, where these electrons are captured by the interface states of Ga_2_O_3_, facilitating rapid charge transfer (Figure S6b, Supporting Information). As LM content increases, the density of interface states rises, promoting more efficient charge transfer and thereby enhancing *p*TENG performance.

More importantly, such an integration of PVDF‐TrFE with LM nanoparticles also enables an increase in dielectric constant of the composite fiber mat, which can benefit the capacitance of the triboelectric electrode and thereby lead to the enhancement in the electric output of the as‐assembled *p*TENG.^[^ ^59^ ^]^ We investigate the impact of LM incorporation on the dielectric constant of LMPT (Figure 2e). The dielectric constant of LMPT exhibited a significantly enhancement, increased from 1.34 to 1.89 at the frequency of 1000 Hz with the content of LM increasing from 0% to 5%. Especially, the dielectric constant of LMPT embedded with 1% LM exhibits a 23% increase in comparison with that of the pristine PVDF fiber mat. Such an enhancement in dielectric constant can be attributed to the sufficient composite dielectric network formed by the incorporated LM nanoparticles (Figure 1c–e), which can significantly improve the overall dielectric performance of the material.^[^ ^40^ ^]^ Further increasing the dielectric constant, in principle, can be achieved by increasing the LM content in the electrospinning dope. However, when the LM content exceeds 1% in LMPT composite, a significant alteration in the fibrous morphology occurs as illustrated in Figures S4 and S5, Supporting Information, where spindles of the aggregation of LM with polymeric matrix are formed. Such morphological changes may lead to the development of local conductive networks, resulting in charge leakage during the contact‐separation of the TENG devices. Consequently, the electrical output of the *p*TENG diminishes, which is reflected in the results of electrical output (Figure 2b–d). The impact of higher LM loading (i.e., larger than 1%) in LMPT on the performance degradation of *p*TENG can also be verified by the capacitance changes of the *p*TENG. The capacitance of the *p*TENG increases from 175 to 190 pF and then decreases to 170 pF as the LM content rises from 0% to 5%. With 1% LM, the capacitance is ≈8% higher than that of pristine PVDF fiber mat, while at 5% LM, it decreases by ≈2.8% (Figure 2f). This capacitance change is mainly attributed to the increased spindles that may reduce the effective contact area (Note S1, Supporting Information) between the triboelectric electrodes, degrading the output performance of the *p*TENG. We further investigate the variation of surface potential of LMPT with respect to LM concentration (Figure S7, Supporting Information). The results show that the surface potential of the LMPT increases with LM content up to 1%, indicating that a higher concentration of LM facilitates more effective storage and retention of triboelectric charges on the surface. Such enhanced surface charge retention is expected to improve electrostatic induction and, consequently, the overall output performance of the *p*TENG. However, when the LM content exceeds 1%, the surface potential decreases, which is consistent with the observed trends in output performance and capacitance. This decline is likely due to the formation of local conductive pathways and increased surface charge leakage caused by LM aggregation, which impedes effective charge accumulation on the surface. These findings further confirm the importance of optimizing LM concentration to modulate the surface electrostatic environment and maximize the triboelectric performance of the *p*TENG.

Upon mechanism analysis, LMPT with 1% LM loading, which provides the optimized output performance, is chosen for the further performance characterization of *p*TENG. The impacts of fiber mat thickness and applied external forces on the electrical performance of the *p*TENG are evaluated. A thinner triboelectric layer can result in a higher electrical performance of the *p*TENG (Figure 2g, Figure S8a–c, Supporting Information). When the LMPT thickness decreases from 175 to 35 µm, the voltage, charge, and current outputs of the *p*TENG increase by ≈52%, 40%, and 39%, respectively. A thinner dielectric layer is beneficial for increasing the capacitance of the *p*TENG. It can be modeled as a series‐connected capacitive pulsed voltage source,^[^ ^59^ ^]^ expressed as 1/C*_p_*_TENG_ = 1/C_LMPT_ + 1/C_spacer_, where C*_p_*_TENG_ is the total capacitance of the *p*TENG, C_LMPT_ is the capacitance of LMPT, and C_spacer_ is the capacitance between the positive and negative triboelectric electrodes. As the thickness of LMPT decreases, C_LMPT_ increases, leading to a higher total capacitance and charge storage of the *p*TENG, thereby enhancing its output performance. Nevertheless, excessively thin layers are prone to damage under external force due to increased friction, compromising the device's durability. Therefore, for wearable *p*TENG fabrication using LMPT, we select a 1% LM‐doped LMPT layer with a thickness of 35 µm as the triboelectric layer to balance performance and stability. Higher electrical output can be achieved upon the application of greater external force to the *p*TENG (Figure 2h, Figure S8d–f, Supporting Information). Increasing the applied external force from 2 to 10 N can lead to the increase in the voltage, charge, and current output of *p*TENG by ≈58%, 44%, and 42%, respectively. Such an enhancement is attributed to the increased external force that effectively enlarges the effective contact area between the two triboelectric electrodes. Furthermore, the *p*TENG also exhibits promising electrical output performance under high‐humidity conditions. Even at a relative humidity of 85%, the open‐circuit voltage, transferred charge, and short‐circuit current still retain 75%, 78%, and 71%, of their valued measured under ambient conditions, respectively (Figure S9, Supporting Information). This moderate decline is mainly due to the adsorbed water molecules on the dielectric surface that result in charge leakage and suppression of charge accumulation. The washing durability of the *p*TENG is also evaluated by following the Standard AATCC 135. The device retains stable performance after 10 washing cycles (Figure S10, Supporting Information). The output voltage, transferred charge, and short‐circuit current exhibited only minor decreases of ≈15%, 13%, and 12%, respectively.

### 2.3 Energy Management for pTENG

**Figure 3:**
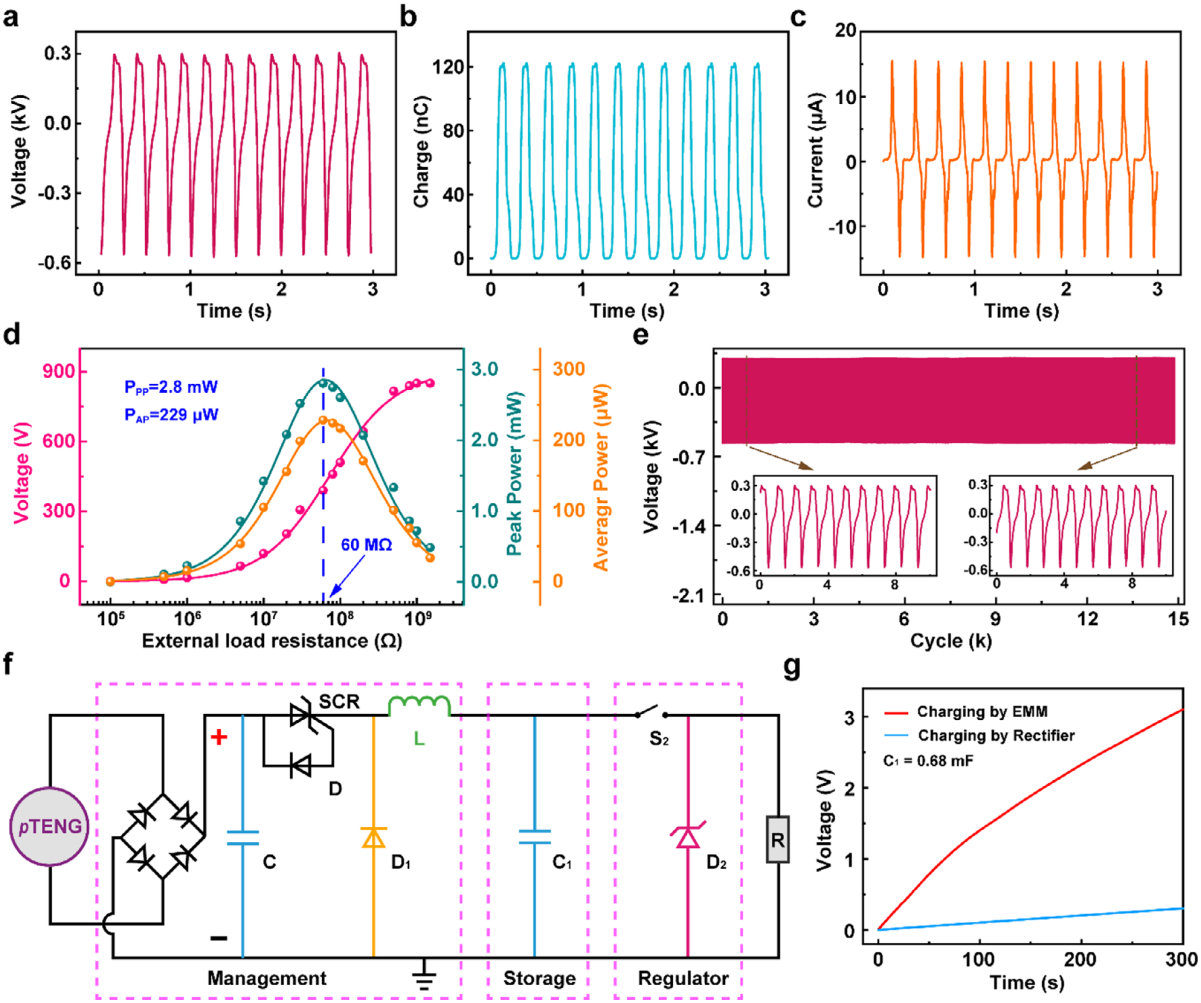
Output performance and efficient energy management of the *p*TENG. a) Open‐circuit voltage, b) Transferred charge, c) Short‐circuit current, and d) Power characteristics of *p*TENG at a working frequency of 4 Hz. e) Voltage output stability of *p*TENG over 15000 cycles. f) Design of energy management module (EMM) for *p*TENG. g) Comparison of the efficiency of charging a capacitor through EMM and a rectifier.

The incorporation of LM nanoparticles into the *p*TENG has led to remarkable improvements in its performance, providing new insights into material optimization for energy harvesting. In particular, with an LMPT area of 25 cm^2^, *p*TENG demonstrates enhanced output characteristics, achieving maximum output voltage of 825 V, charge of 120 nC, and current of 15 µA (**Figure** **3**a–c). More importantly, *p*TENG based on LMPT can achieve a peak power of 2.8 mW and an average power of 229 µW under a 60 MΩ external load (Figure 3d). This voltage output performance can be well retained over 15000 operational cycles (Figure 3e), which is especially advantageous for long‐term energy harvesting applications.

However, the pulsed AC output voltage of the *p*TENG cannot directly power wireless electronic devices that typically require a stable and continuous voltage supply. Though several electronic switch (E‐switch) strategies have been developed for wearable TENG devices, most of them rely solely on rectifier bridges to manage the pulsed output of TENGs (Table S2, Supporting Information).^[^ ^60^, ^61^, ^62^, ^63^, ^64^ ^]^ These approaches, however, are inadequate for maximizing energy harvesting, since rectifier bridges cannot dynamically match the high output impedance of the TENG, leading to inefficient charge transfer. Furthermore, these approaches generally lack effective regulation or control over the harvested energy, resulting in uncontrolled voltage rise and significant energy leakage through the load circuit. To solve this challenge, an Energy Management Module (EMM) is specifically designed for *p*TENG as a unified E‐switch, which consists of a management unit, a storage unit, and a regulator unit (Figure 3f). The management unit, comprising of a rectifier bridge, a capacitor (C), a thyristor (SCR), a diode (D), an inductor (L), and a parallel freewheeling diode (D_1_), is used to regulate the voltage and impedance of the *p*TENG. The physical layout for the management unit is illustrated in Figure S7 (Supporting Information). When the output voltage of the *p*TENG, upon passing through the rectifier bridge, exceeds the reverse breakdown voltage of the diode D (i.e., 200 V in this design), SCR remains turned on (Figure S11a‐i, iii, Supporting Information). At this stage, the energy generated by the *p*TENG accumulates in inductor L as the human body continues mechanical motion and diode D_1_ remains closed. When the output voltage of the *p*TENG, upon passing through the rectifier bridge, is below the reverse breakdown voltage of the diode D, SCR remains turned off (Figure S11a‐ii, iv, Supporting Information). The electrical energy generated by the pTENG is then released from inductor L to the storage unit (i.e., capacitor C_1_), while diode D_1_ remains open, forming an energy conversion loop. The *p*TENG continuously harvests mechanical energy from human motion, leading to the SCR repeatedly switching on and off, and subsequently converting the harvested energy into electrical energy with a maximum efficiency of 50%. The converted energy is then stored in the storage unit (C_1_), thereby increasing the voltage of C_1_. To further address the need for stable DC power in electronics, we incorporate a voltage regulator unit that consists of a switch S_2_ and regulator D_2_ into EMM, which can regulate the electrical energy from capacitor C_1_ into a stable DC electricity. Briefly, Switch S_2_ comprises a series of monitor chips (Q_1_ – Q_4_), resistors (R_1_ and R_2_), Zener Diode (D_3_), and capacitors (C_2_ – C_6_), as well as a triode (Tr_1_). The detailed circuit design and physical layout for the voltage regulator unit are illustrated in Figure S12 (Supporting Information). When the voltage of capacitor C_1_ reaches the threshold of 4.4 V (i.e., predetermined voltage value sufficient to power the wireless wearable devices demonstrated in this work), switch S_2_ activates, achieving 2.5 V of DC electricity (Figure S13, Supporting Information). The energy released by capacitor C_1_ during the discharge phase can be calculated using the equation $J=\frac{1}{2}\;C_{1}\left(V_{initial}^{2}-V_{final}^{2}\right)$, where *J* is the energy released by capacitor  C_1_, *C*_1_ is the capacitance of C_1_ (i.e., 0.68 mF), *V_initial_* is the threshold voltage that triggers the activation of switch S_2_ (i.e., 4.4 V), and *V_final_* denotes the discharge‐end voltage of the storage capacitor when switch S_2_ is deactivated (i.e., 2.5 V), yielding ≈4.457 mJ. Such a voltage value and the amount of energy generation are sufficient to fully power a wireless sensing system, such as the temperature sensing and signal transmission demonstrated in our proof‐of‐concept system described in below section, which requires only 4.45 mJ for one complete sensing and transmission cycle. Compared with the conventional rectifier‐only designs, this unified EMM design can significantly enhance the energy management efficiency and maintain a stable 2.5 V output for *p*TENG. While *p*TENG managed only by a rectifier bridge, which is a commonly adopted practice for regulating the output of TENG, can only charge capacitor C_1_ (0.68 mF) to 0.29 V in 300 s, *p*TENG with EMM can efficiently charge C_1_ to 3.1 V within the same time frame (Figure 3g). This comparison demonstrates a ten‐fold increase in efficiency by using the developed EMM, clearly demonstrating its advantages for wearable TENG applications.

### 2.4 *p*TENG for Self‐Powered Wireless Temperature Monitoring

**Figure 4:**
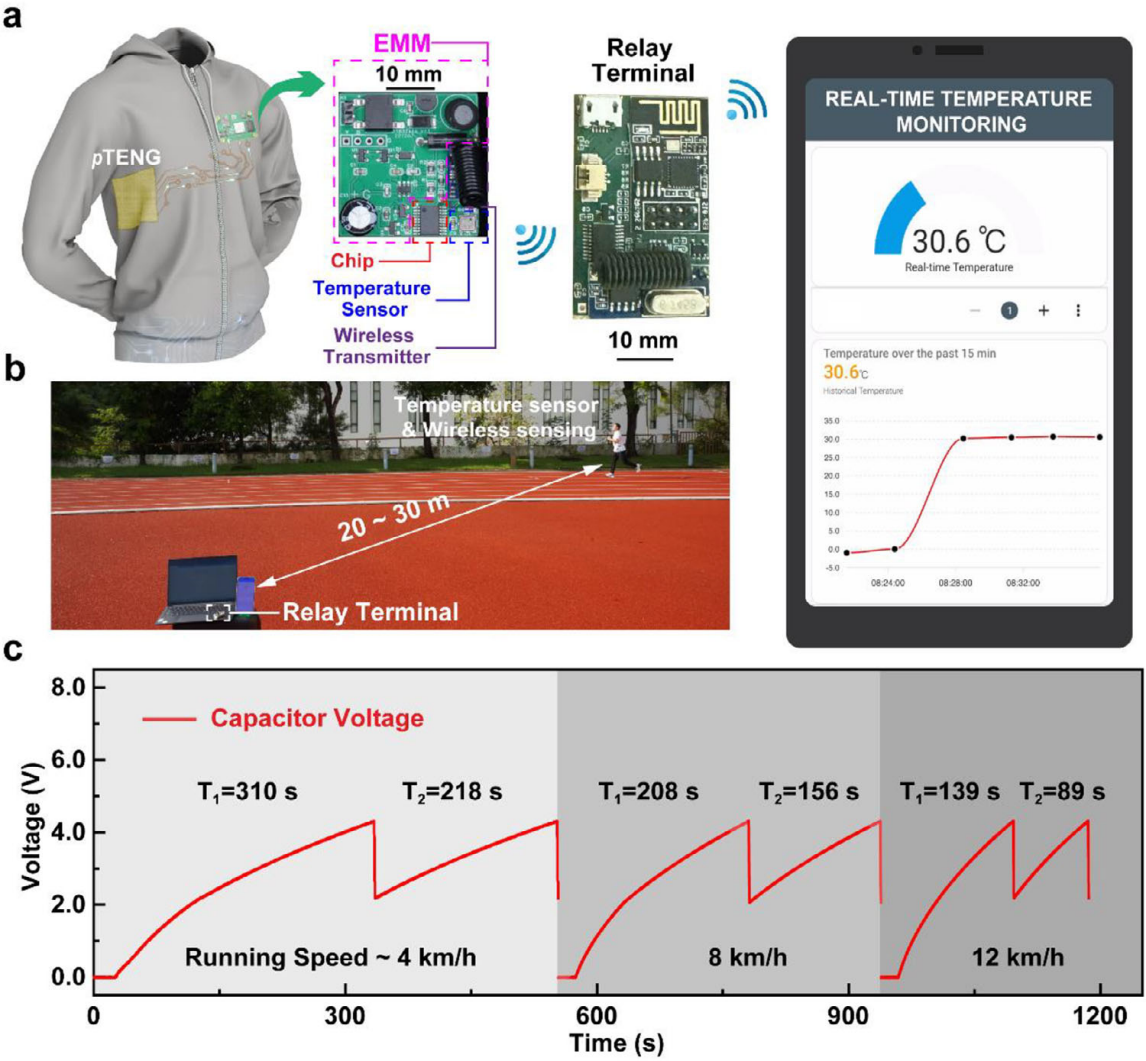
Application of the self‐powered system based on *p*TENG for wireless temperature monitoring. a) Working process of the self‐powered system based on *p*TENG for wireless temperature monitoring. b) Demonstration of wireless temperature monitoring during running. c) Voltage changes of the energy storage module in EMM at different running speeds (T_n_: time required to charge the energy storage module in EMM to the predetermined voltage (i.e., 4.4 V, _n_ is the number of charges).

The permeable and all‐textile‐based *p*TENG, showcasing exceptionally high power output, can seamlessly integrate into a wearable format. This integration, when coupled with EMM, allows for the effective harvesting of human motion energy and the efficient conversion into electric energy for self‐charging wearable electronics. As proof of concept, we develop a self‐powered garment integrating the LMPT‐based *p*TENG with EMM, the temperature sensing system, and wireless transmission. This self‐powered system operates by solely relying on the biomechanical energy converted by the *p*TENG, without the need for any external power supply. The *p*TENG is encapsulated with a layer of thermoplastic polyurethane (TPU) fiber mat for waterproof and protection purpose, and then fixed on the lateral sides of the T‐shirt. Notably, the TPU‐encapsulated *p*TENG still maintains a high moisture permeability of 600 g m^−^^2^ day^−1^ (Figure S14, Supporting Information), meeting the requirement for insensible perspiration and thermoregulation of human skin (300–600 g m^−^^2^ day^−1^).^[^ ^65^, ^66^ ^]^ The *p*TENG is subsequently linked to EMM to create an energy‐harvesting module circuit. This circuit can supply electricity to the wireless temperature sensing system that consists of a temperature sensor, a chip for processing the collected temperature data, and a wireless transmitter for transmitting the data to the relay terminal (**Figure** **4**a). The relay terminal is an independent external circuit, separate from both the wireless temperature sensing system and its energy supply circuit (i.e., *p*TENG and its linked EMM). It consists of a chip, a wireless receiver, and a Wi‐Fi module, which is powered by a battery. This setup enables the relay terminal to upload the received temperature information to the cloud through Wi‐Fi technology. Afterward, the information is received and real‐time displayed on a monitoring terminal (i.e., smartphone in this demonstration), through which a self‐powered wireless temperature monitoring system for exercise is constructed. Detailed fabrication procedures as well as the parameters of each module in the system are described in the experimental section and summarized in Figures S15 and S16, and Tables S3–S5 (Supporting Information).

When worn by the runner, this self‐powered T‐shirt utilizes the integrated *p*TENG to harness the kinetic energy during running. The natural swinging motion of the arm during walking or running periodically compresses and releases the device, resulting in repeated contact and separation between the two triboelectric layers. This vertical mechanical deformation enables the *p*TENG to generate continuous electrical output under the contact‐separation working mode without the need for intentional external force. The harnessed energy is then converted into electrical energy and stored in the storage module of EMM, subsequently supplying the required electricity to the wireless sensing integrated system. Upon reaching the voltage threshold of the energy storage module in EMM (i.e., 4.4 V, the predetermined voltage value sufficient to power the temperature sensing and wireless transmission (power consumption: ≈22.5 mW), the voltage is automatically regulated to a stable 2.5 V by the voltage regulator unit of EMM, which is suitable to power the wireless sensing system. Notably, the energy harvested by *p*TENG is sufficient to allow for the wireless and real‐time transmission of the collected temperature data to the relay terminal within a distance of 20–30 m (Figure 4b; and Video S1, Supporting Information). Once the system's electrical energy is depleted for the temperature sensing and wireless transmission, it can be replenished by continuing collecting the energy from running to reach the predetermined voltage again. This re‐energizing process enables self‐powered and continuous temperature monitoring during the exercise. We investigate the relationship between charging efficiency and running speed by performing treadmill‐based tests. The output voltage of the *p*TENG at different running speeds ranging from 4 to 12 km h^−1^ (Figure S17, Supporting Information) remains stable at ≈600 V across all tested speeds, indicating that the *p*TENG can reliably generate high‐voltage output regardless of motion intensity. While the output voltage is independent of the running speed, the power supply capability of the *p*TENG is closely related to the efficiency of the energy harvesting from the human motion. Specifically, the faster the running speed, the greater the mechanical energy generated, leading to higher efficiency in energy harvesting by the *p*TENG. Consequently, when the running speed increases from 4 to 12 km h^−1^, the time required to charge the energy storage module in the EMM to 4.4 V is reduced by ≈55%, demonstrating the enhanced charging performance under faster motion. Importantly, during walking and running, occasional movements, such as jumping, arm waving, or even resting, do not affect the storage of harvested energy. The output voltage will continue to increase with ongoing walking for running motions until the required voltage threshold is reached to activate the sensor operation (Figure S18, Supporting Information), allowing for the reliable operation of the temperature sensor. After consuming the power for signal transmission, running at the speed of 12 km h^−1^ can complete the energy replenishment within 89s, which is 43% faster than running at the speed of 8 km h^−1^ and 60% faster compared to running at the speed of 4 km h^−1^ (Figure 4c).

## 3 Conclusion

In conclusion, we have introduced a permeable triboelectric nanogenerator (*p*TENG) with a remarkable energy output exceeding 35 V cm^−^^2^, ensuring the ability to power a wireless sensing system without hindering skin perspiration. Such high output of this *p*TENG is attributed to the efficient composite dielectric fibrous network formed by uniformly distributed LM nanoparticles in the electrospun LMPT, which significantly enhances the dielectric constant of the triboelectric electrode. Incorporated by a tailor‐designed energy management module, *p*TENG can achieve a charging speed 10 times faster compared to the counterpart with commonly adopted rectifier. As a proof of concept, a smart garment integrated with a self‐powered wireless temperature sensing system is constructed, which is comprised of the newly developed LMPT‐based *p*TENG, the energy management module, the temperature sensor, and the wireless transmitter. By continuing harvesting human motion energy by the *p*TENG during the exercise, such a self‐powered system enables the real‐time tracking and display of the temperature. This integration, by incorporating high‐output energy harvester, energy management module, and active wireless sensing technologies, not only reduces the reliance on external power source but also promotes the real‐time and wireless health monitoring, showcasing the high application promise in body area networks for enhanced personalized healthcare and well‐being monitoring.

## 4 Experimental Section

### Fabrication of LMPT

All chemicals were employed in the experiments without undergoing additional purification. The LMPT is prepared via electrospinning of an electrospinning dope mixed with the suspension of LM nanoparticles and the PVDF‐TrFE solution. The suspension of LM nanoparticles was formed by probe sonication (TL‐250Y, Jiangsu Tenlin Instrument Co., Ltd., China) of bulk LM in a solvent. Briefly, bulk LM (Ga: In (weight ratio: 15.1:4.9), 16 °C, Dingguan Metal Co., Ltd., China) with different masses (0.0400, 0.0800, 0.1608, 0.4082, and 0.8380 g) were added to 4 g solvent mixture of DMF (N, N‐Dimethylformamide, 99.5%, DUKSAN) and acetone (99.8%, RCI Labscan Limited) (weight ratio: 3:1) in a 20 mL glass container. The container was then placed in an ice bath, where the temperature was maintained at 0–3 °C during the probe sonication process. The probe sonication is conducted at 80% power under a burst mode (on/off, 2 s) for 2 h. The PVDF‐solution was prepared by dissolving PVDF‐TrFE pellets (Xinsuyuan Plastic Technology Co., Ltd., China) into the solvent mixture of DMF and acetone at a concentration of 13.66% wt. by magnetic stirring for 2 h at 75 °C. To prepare the electrospinning dope containing LM and PVDF‐TrFE, the LM suspension and PVDF‐TrFE solution were mixed at weight ratios of (0.339, 0.342, 0.349, 0.370, and 0.406):1 (Figure S1, Supporting Information). LMPT composite fiber mat was then fabricated by the electrospinning of the dope containing LM and PVDF‐TrFE at a voltage of 14 kV (TL‐Pro, Shenzhen Tongli Micro‐Nano Technology Co., Ltd., China). The collection distance and a flow rate of the dope were set as 15 cm and 2.5 mL h^−1^, respectively. The humidity and temperature inside the electrospinning chamber are maintained at 55 ± 5% and 35 ± 5 °C, respectively. The electrospun fiber mat was collected on the Ni fabric and subsequently dried at 80 °C overnight. For comparison, PVDF‐TrFE fiber mat without the incorporation of LM was fabricated with the same electrospinning conditions.

### Assembly of *p*TENG

*p*TENG was assembled by stacking two pieces of triboelectric electrode fabrics, including one electrospun LMPT fiber mat on the Ni fabric as the bottom electrode (negative triboelectric layer) and one Ni fabric as the top electrode (positive triboelectric layer). To enhance the waterproof and stability of the triboelectric electrodes, a layer of TPU fiber mat was respectively electrospun on the outer sides of the LMPT electrode and Ni fabric electrode before the assembly. Finally, the obtained electrospun films were subsequently dried at 80 °C overnight. After then, these two TPU‐encapsulated electrodes were connected using an elastic Kapton film to form a contact‐separation structured *p*TENG.

### Materials Characterization and Electric Measurement

The morphologies the electrospun fiber mats were investigated by Field Emission Scanning Electron Microscope (SEM, Tescan MAIA3) and Field Emission Transmission Electron Microscope (TEM, JEOL JME‐2100F). The elemental characterization for the fiber mat was carried out by the energy dispersive spectroscopy in SEM. The tensile stress‐strain curves of the electrospun films were obtained using an Instron 5599 universal testing system. The moisture permeability test of the samples was conducted using the cup method in accordance with the textile standard E96/E96M‐13. The moisture vapor transmission rate (g/m^2^/day) was determined by measuring the weight loss of water vapor from a cup, which was securely covered with the test sample (test duration: 72 h). The air permeability test was conducted using an MO21S air permeability tester (SDL Americ, Inc.) in accordance with the ASTM D737‐08 standard. The thickness of the electrospun films was regulated mostly by electrospinning time and characterized by high‐precision thickness gauge (SYA221176471). To evaluate washing durability, a standardized washing test was carried out following the Standard AATCC 135 (Delicate program), in which the *p*TENG samples were placed in a washing bag and washed with a 1.8 kg fabric load for 45 min. This condition was designed to simulate practical laundering scenarios and examine the stability of the *p*TENG's output performance after washing.

The open‐circuit voltage and charge of the *p*TENG were measured using a programmable electrometer (Keithley 6517B) connected with a high‐voltage probe (HVP‐40). The surface potential of the *p*TENG was characterized by using an electrostatic voltmeter (Trek 347). The LMPT sample was mounted on a motorized three‐axis stage. By changing the position of the sample beneath the probe, the local surface potentials were recorded at multiple positions, and the average value along with the standard deviation was calculated to evaluate the overall surface potential distribution. The cycling stability test of the *p*TENG was conducted using a fatigue testing machine (LT‐1, Dongguan Huaguo Precision Instrument Co., Ltd., China). The force applied to the *p*TENG was measured using a spoke‐type load cell (JLBU‐1). The capacitance of *p*TENG and LMPT was measured using an LCR meter (Keysight, E4980A). The relative permittivity (ε_*r*_) of the LMPT is determined using the equation ε_*r*_ = *t_LMPT_*  · *C_LMPT_*/*S_LMPT_*/ε_0_, where *t_LMPT_* is the thickness of LMPT, *C_LMPT_* is capacitance of LMPT, *S_LMPT_* is the measure area of LMPT, and ε_0_ (8.854 × 10^−12^ F/m) is the vacuum dielectric constant.

### Fabrication of Energy Management Module and the System Integration

The energy management module first uses a rectifier bridge, a ceramic capacitor, a thyristor, a diode, an inductor, and a parallel freewheeling diode as a management unit to regulate the voltage and impedance of the *p*TENG. Then, a storage capacitor was used to store the energy generated by the *p*TENG. Finally, switch S_2_, comprising a series of monitor chips (Q_1 –_ Q_4_), Zener Diode (D_3_), resistors (R_1_ and R_2_), capacitors (C_2 –_ C_6_), and a triode (Tr_1_), were employed to regulate the electrical energy from the storage capacitor (C_1_), ensuring a stable DC output. The working current of S_2_ is ≈4  µA at a maximum working voltage of 4.4 V and its operating duration is ≈0.17 s (Figure S19, Supporting Information). As such, the maximum energy consumption of S_2_ is ≈2.992 µJ, corresponding to a power consumption of ≈17.6 µW. This extremely low energy requirement ensures that majority of the harvested energy stored in the capacitor C_1_ could be effectively utilized by the wireless sensing components with minimal loss during S_2_ operation, demonstrating the high energy efficiency of the system design and the feasibility of self‐powered operation. Table S3 (Supporting Information) provides a detailed summary of the component parameters of the energy management module.

The wireless sensing integrated system primarily consists of an energy management module as the stable DC power supply, a temperature sensor, a chip, and a wireless transmitter. The chip for temperature signal processing was programmed by the software Keil. This system enables the acquisition and wireless transmission of temperature signals, consuming ≈4.45 mJ of energy (≈22.5 mW power) during the process. Figure S11 (Supporting Information) presents the detailed circuit design diagram, while Table S3 (Supporting Information) provides a comprehensive summary of the component parameters in the wireless sensing integrated system.

The relay terminal module mainly consists of a wireless receiver, a chip, a voltage stabilizer, a lithium‐ion battery, and a Wireless Fidelity (Wi‐Fi) module, which could receive data from the wireless transmitter, upload it to the cloud server, and display the data in the real‐time monitoring terminal (i.e., smartphones and computers in this work). The chip for temperature signal processing was programmed by the software Keil. A 400 mAh lithium‐ion battery, with a nominal voltage of 3.7 V, could directly power the entire relay terminal module. Figure S16 (Supporting Information) presents the detailed circuit design diagram, while Table S5 (Supporting Information) provides a comprehensive summary of the component parameters in the relay terminal module.

### Statistical Analysis

The output data of the *p*TENG were directly collected from the electrometer (Keithley 6517B) without the need for further transformation or normalization. Data were presented as mean ± standard deviation, typically based on five independent measurements. All electrical output parameters, including open‐circuit voltage, short‐circuit current, and transferred charge, were measured at least three times under identical conditions. All data processing was performed using Origin and Microsoft Excel.

## Conflict of Interest

The authors declare no conflict of interest.

## Author Contributions

Y.Q. performed conceptualization, data curation, formal analysis, investigation, methodology, software, validation, visualization, wrote the original draft, wrote, reviewed, and edited the draft, and provided resources. J.J. performed methodology and software. F.C. and J.Z. performed data curation. J.L. performed investigation. J.F. and Y.Y. performed formal analysis. Y.D. performed methodology. Z.Z. provided resources. Q.H. performed project administration and supervision.

## Supporting information



Supporting Information

Supplemental Video 1

## Data Availability

The data that support the findings of this study are available in the supplementary material of this article.
