# Supplementary material for: A Permeable Triboelectric Fiber Mat with 35 V cm−2 Voltage Output for Wearable Wireless Sensing Electronics
Source: Small. 2025 Jul 2;21(34):2504556. doi: 10.1002/smll.202504556 (PMC12393031; doi:10.1002/smll.202504556)
Supplement: Supplementary file 1 — Supporting Information [file SMLL-21-2504556-s001.docx]

Supporting Information

# A Permeable Triboelectric Fiber Mat with 35 V/cm^2^ Voltage Output for Wearable Wireless Sensing Electronics

Youchao Qi, Jinxing Jiang, Fan Chen, Junhua Zhou, Jiaheng Liang, Jingjing Fu, Yongqiang Yang, Yichun Ding, Zijian Zheng, Qiyao Huang*

Y. Qi, J. Jiang, Y. Ding, Q. Huang

School of Fashion and Textiles, The Hong Kong Polytechnic University, Hong Kong SAR, P. R. China
E-mail: qi-yao.huang@polyu.edu.hk

F. Chen, J. Zhou, J. Liang, J. Fu, Y. Yang, Z. Zheng
Department of Applied Biology and Chemical Technology, Faculty of Science, The Hong Kong Polytechnic University, Hong Kong SAR, P. R. China

Z. Zheng, Q. Huang

Research Institute for Intelligent Wearable Systems, The Hong Kong Polytechnic University, Hong Kong SAR, P. R. China

Z. Zheng

Research Institute for Smart Energy, The Hong Kong Polytechnic University, Hong Kong SAR, P. R. China

Z. Zheng

PolyU-Wenzhou Technology and Innovation Research Institute, Wenzhou, Zhejiang, P. R. China

### Note S1. Effective contact area analysis in *p*TENG

This capacitance decrease is mainly attributed to the reduction in effective contact area, which is calculated to decrease from 5.16 cm² to 3.56 cm² as the LM content increased from 0% to 5%. The effective contact area is estimated using the classical parallel-plate capacitance equation, C*_p_*_TENG_ = ε_₀_ε_r_S/d, where C*_p_*_TENG_ is the capacitance of the *p*TENG, ε_₀_ (8.85 × 10⁻¹² F/m) is the vacuum permittivity, ε_r_ is the relative dielectric constant of the triboelectric layer, S is the effective contact area, and d (35 μm) is the dielectric layer thickness. Consequently, the observed decrease in capacitance is primarily attributed to the increased presence of spindle-like structures, which reduce the effective contact area between the triboelectric electrodes and thereby degrade the output performance of the *p*TENG. In addition, the aggregation of LM within these spindles may lead to interfacial charge leakage during the contact-separation process, further contributing to the decline in device performance.


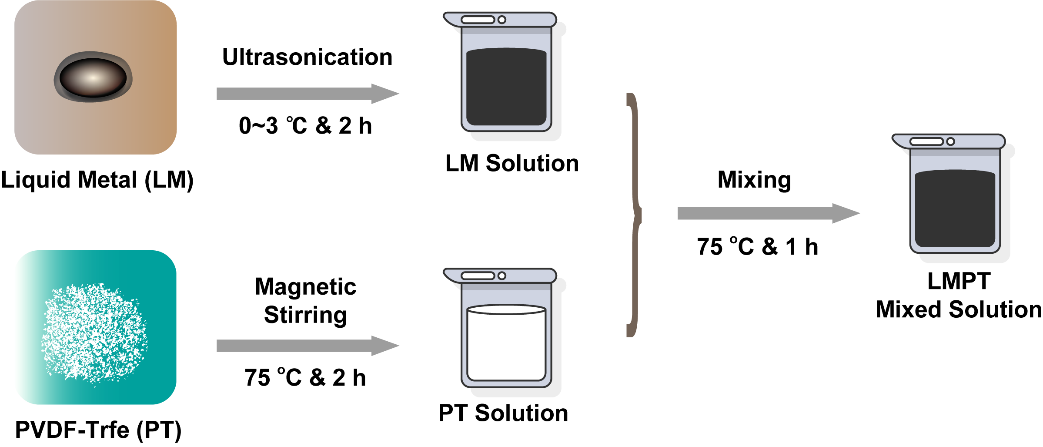


### Figure S**1.** Schematic illustration showing the preparation of the electrospinning dope for LMPT.


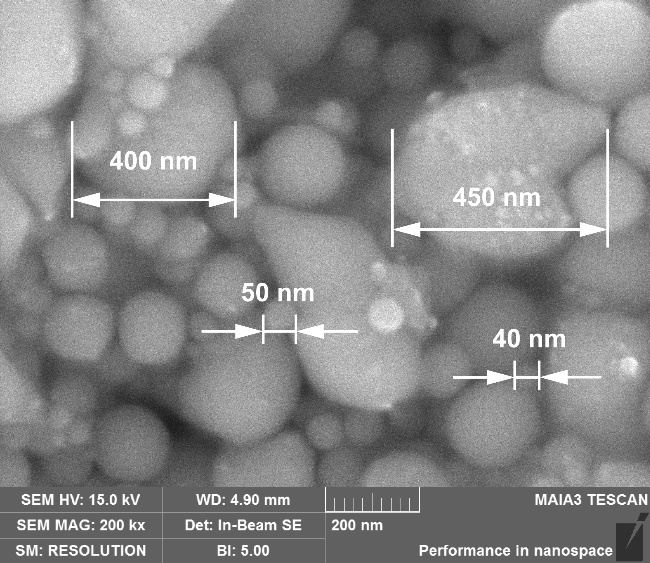


### Figure S**2.** Scanning electron microscopy (SEM) image showing the size of LM nanoparticles.

**
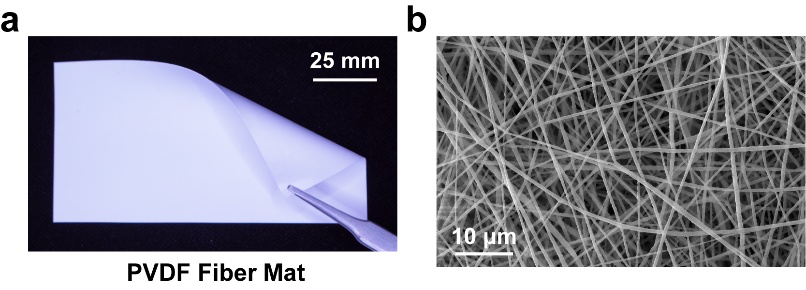
**

### Figure S**3.** Optical image and SEM image of the PVDF fiber mat: a) Optical image; b) SEM image.

**
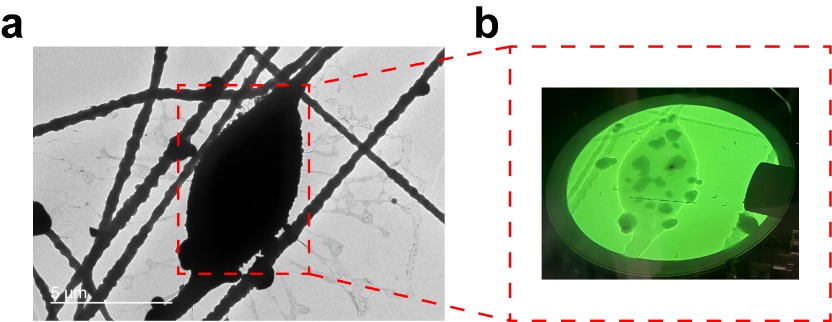
**

### Figure S**4.** LM particles aggregate to form spindles. a) TEM image of the spindle. b) Optical image of LM in the spindle.


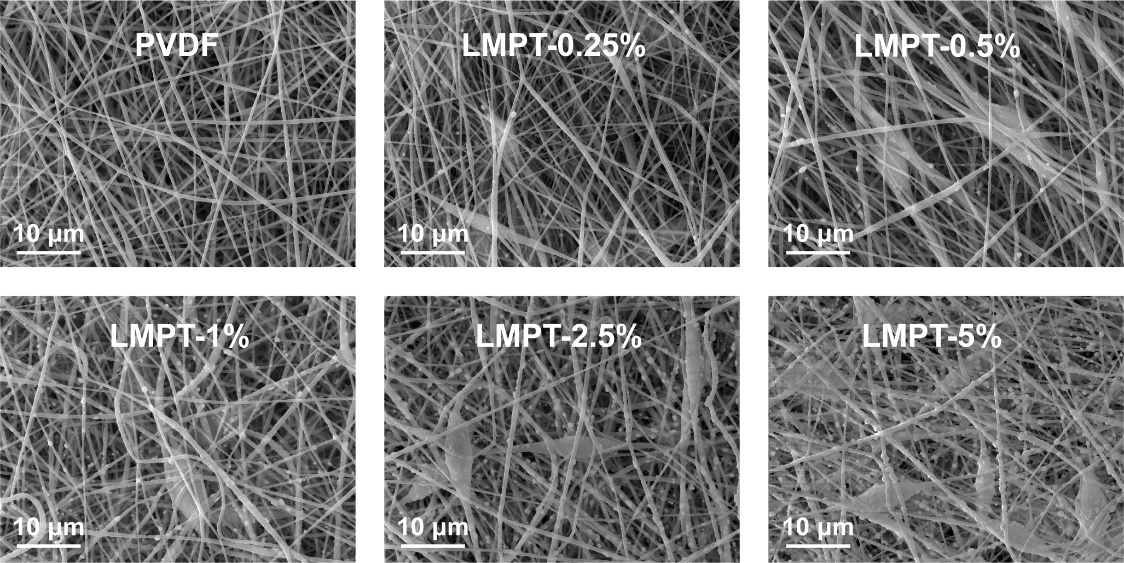


### Figure S**5.** SEM images of LMPTs with varying LM content.


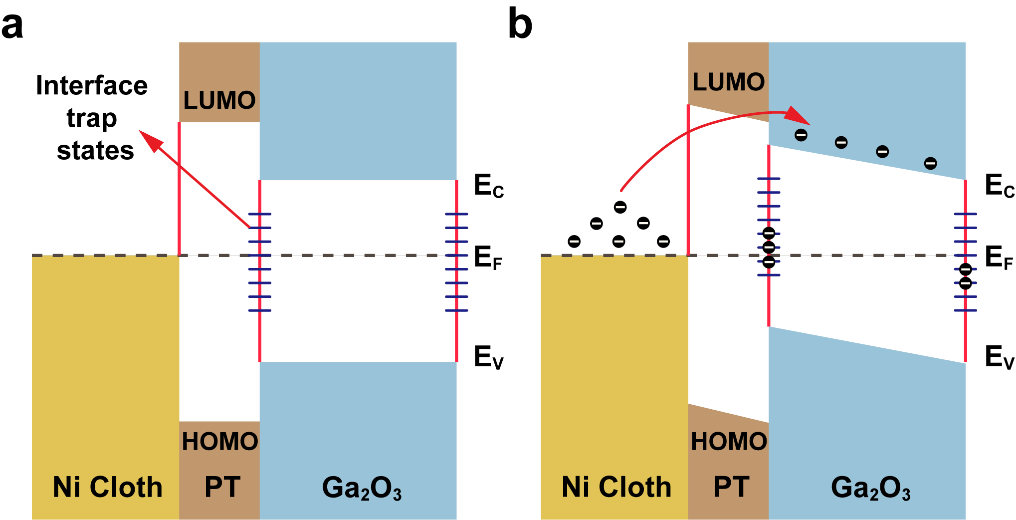


### Figure S**6.** Schematic illustration of Ga₂O₃ band structure and charge trapping: a) Before and b) After charge accumulation on the LMPT surface.

**
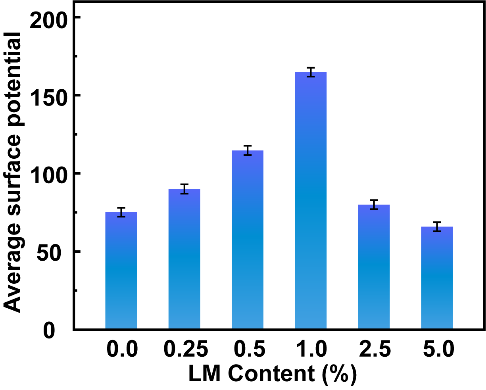
**

### Figure S**7.** Surface potential of LMPT composites with different LM contents (the data were from the mean of four hundred sets of experimental data, and the error bar was taken from the standard deviation).

**
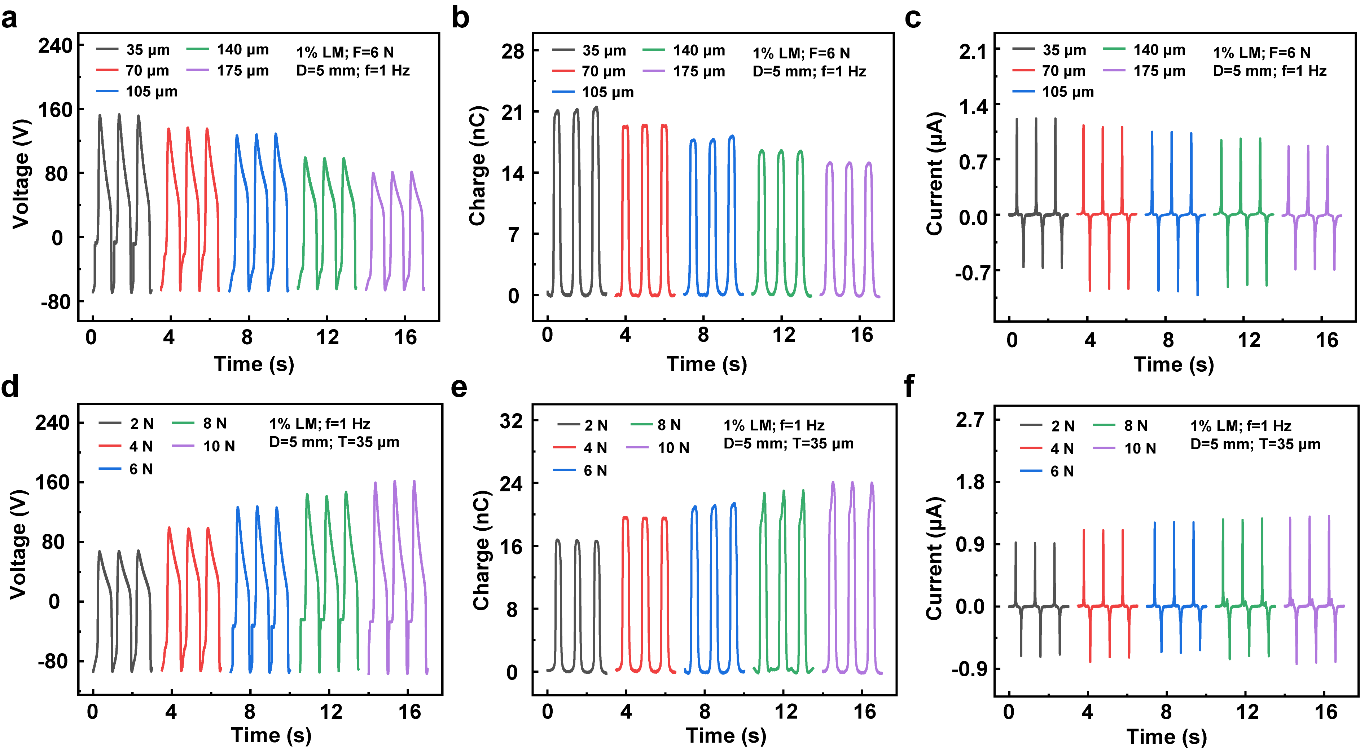
**

### Figure S**8.** Effect of LMPT film thickness and external force on the electrical output of *p*TENG**.** a) Open-circuit voltage, b) Transferred charge, and c) Short-circuit current of the *p*TENG at varying LMPT thicknesses. d) Open-circuit voltage, e) transferred charge, and f) short-circuit current of the *p*TENG at different contact force.


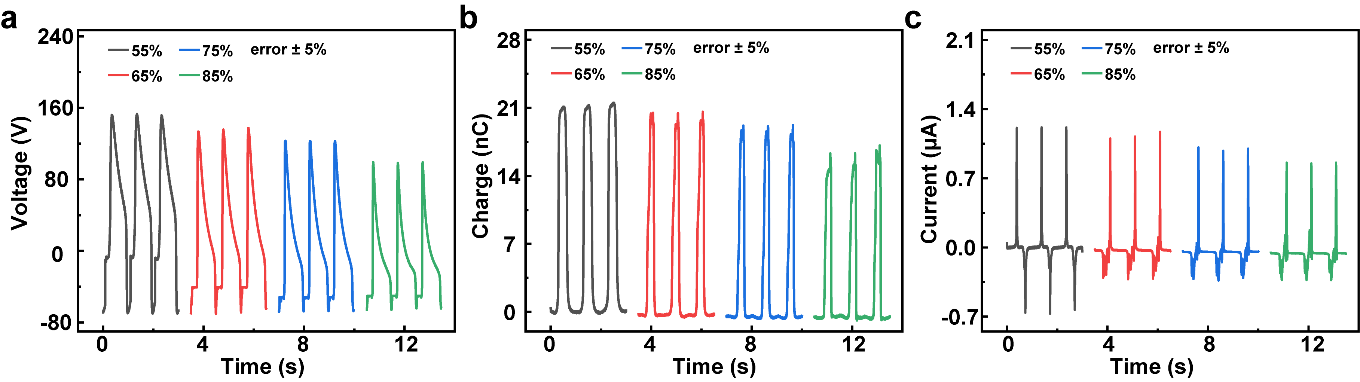


### Figure S**9.** Effect of relative humidity on the electrical output of the *p*TENG**:** a) Open-circuit voltage, b) Transferred charge, and c) Short-circuit current under different humidity levels.


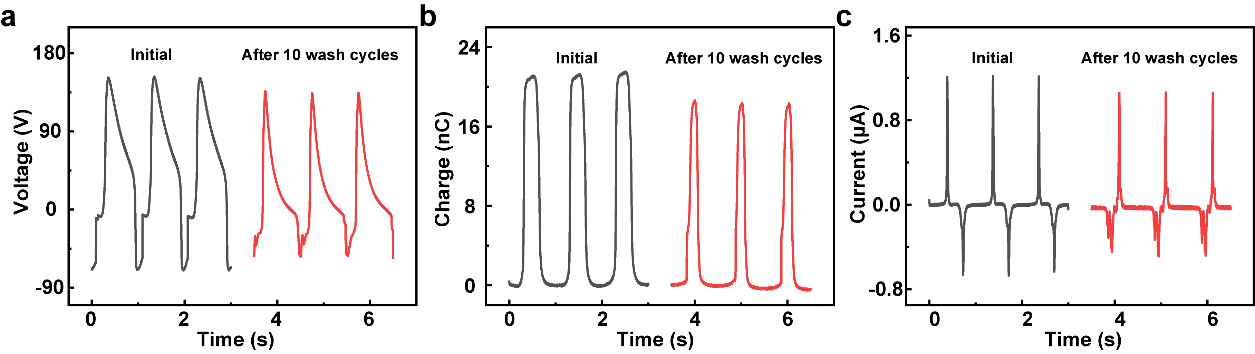


### Figure S10. Output performance of the *p*TENG after 10 washing cycles, showing only slight degradation in voltage a), charge b), and current c) outputs.

**
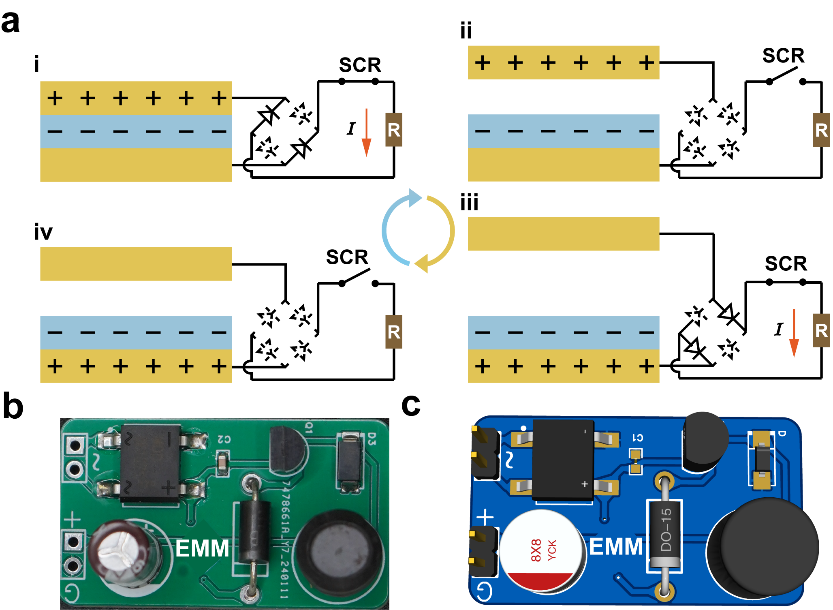
**

### Figure S**11.** Working mechanism and physical layout of management unit. a) Working mechanism of management unit for maximizing the output of the *p*TENG. b) Optical image of management unit. c) 3D model of management unit.

**
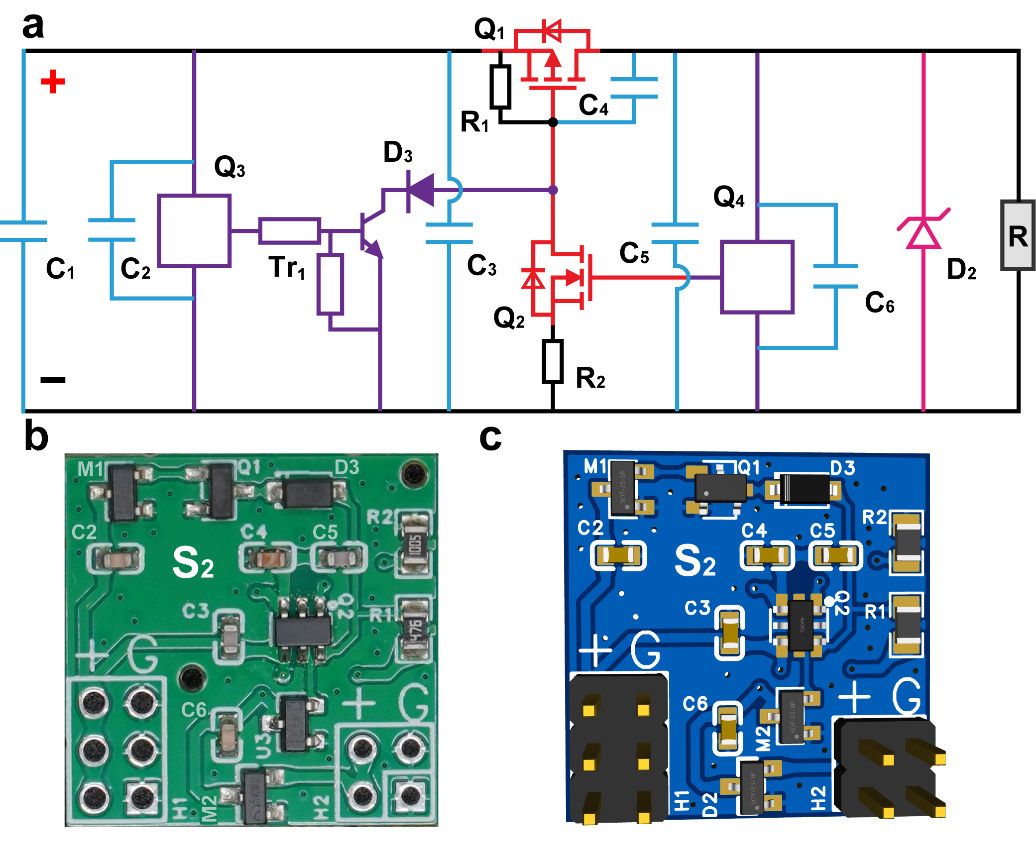
**

### Figure S**12.** Design scheme of switch S_2_. a) Design of switch S_2_. b) Optical image of switch S_2_. c) 3D model of switch S_2_.

**
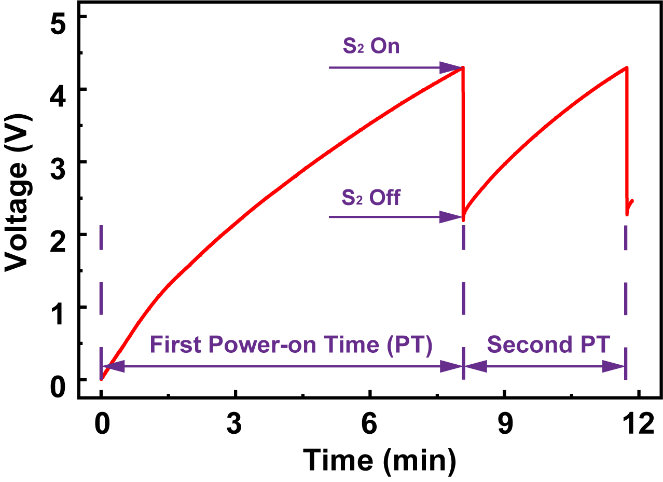
**

### Figure S**13.** Voltage of capacitor C_1_ regulated by switch S_2_.

**
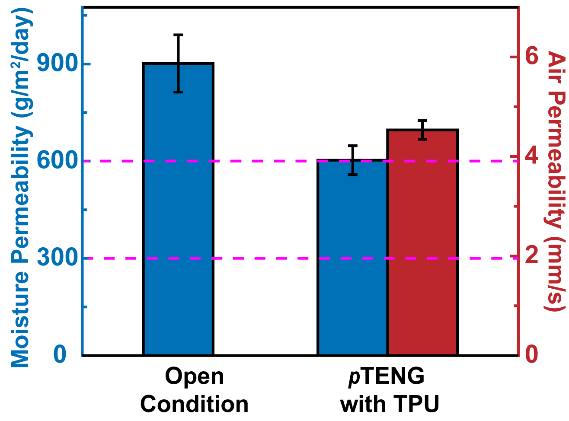
**

### Figure S**14.** Moisture and air permeability of *p*TENG encapsulated with electrospun TPU fiber mat.


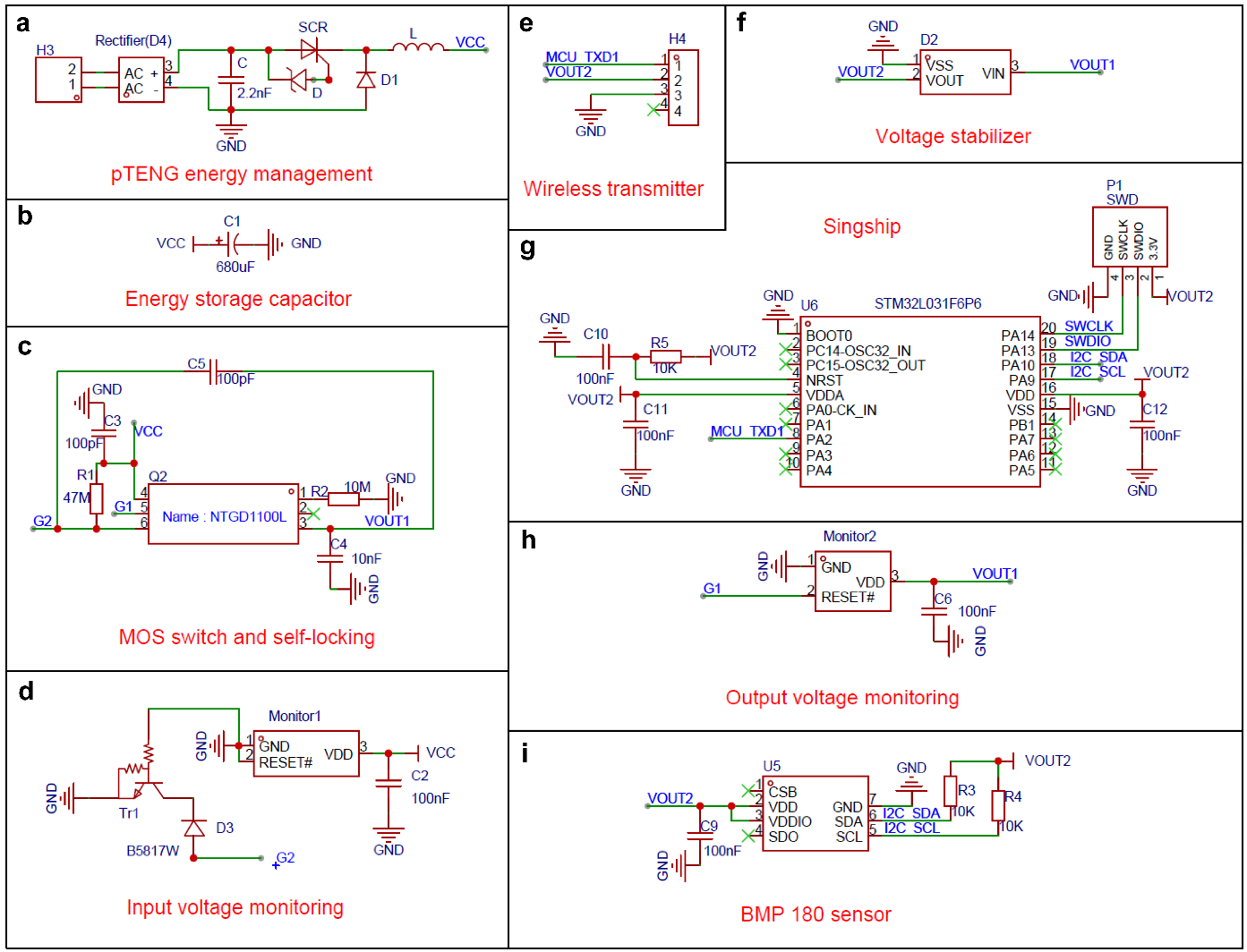


### Figure S15. Circuit design of self-powered wireless sensing node. a) *p*TENG energy management circuit. b) Energy storage circuit. c) MOS switch and self-locking circuit. d) Input voltage monitoring circuit. e) Wireless transmitter circuit. f) Voltage stabilizer circuit. g) Singlechip peripheral circuit. h) Output voltage monitoring circuit. i) Temperature sensing circuit.


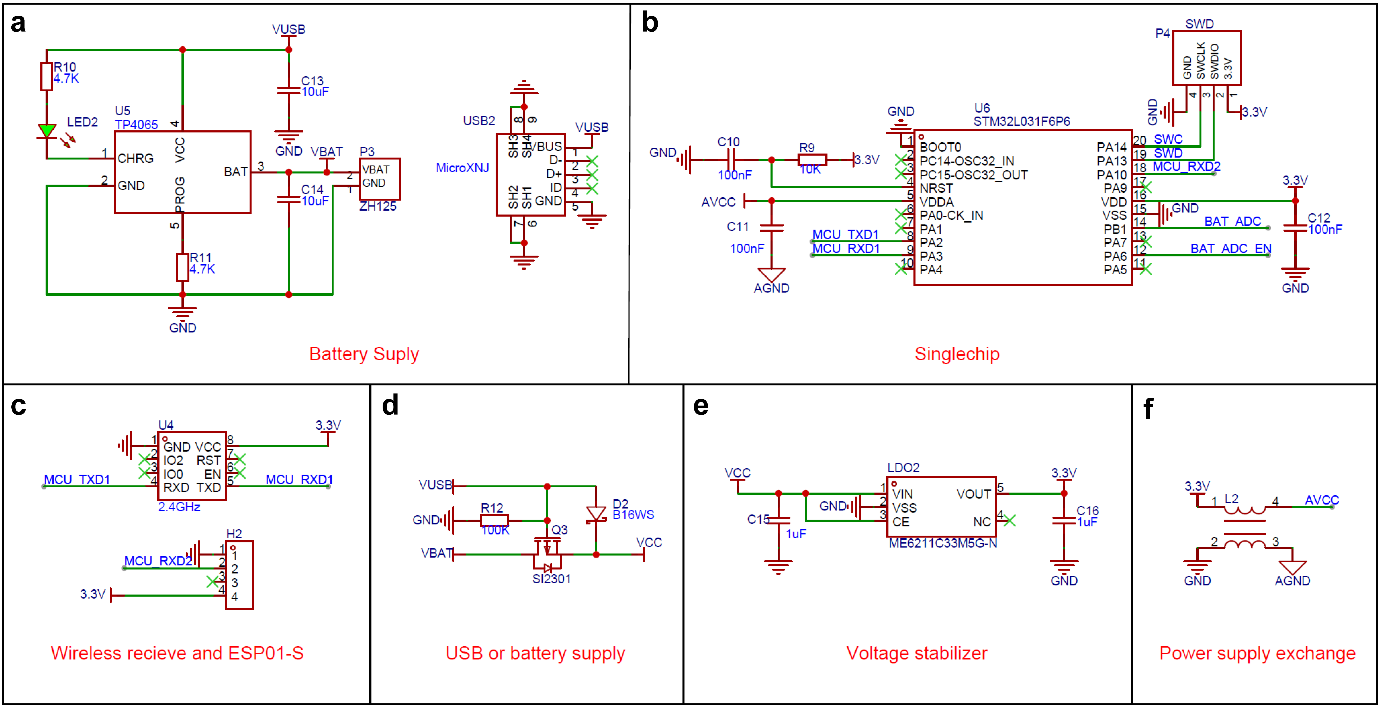


### Figure S16. Circuit design of the Wi-Fi relay terminal. a) Battery supply circuit. b) Singlechip peripheral circuit. c) Wireless receiver and ESP01-S circuit. d) USB or battery supply circuit. e) Voltage stabilizer circuit. f) Power supply exchange circuit.


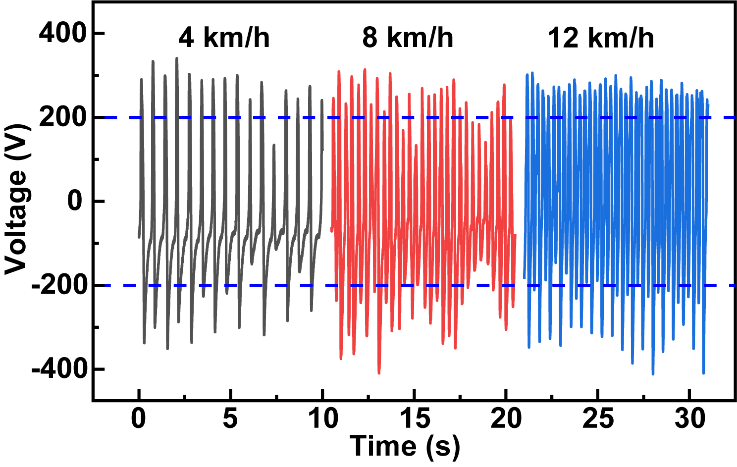


### Figure S17. Voltage output of the *p*TENG at running speeds ranging from 4 to 12 km/h.


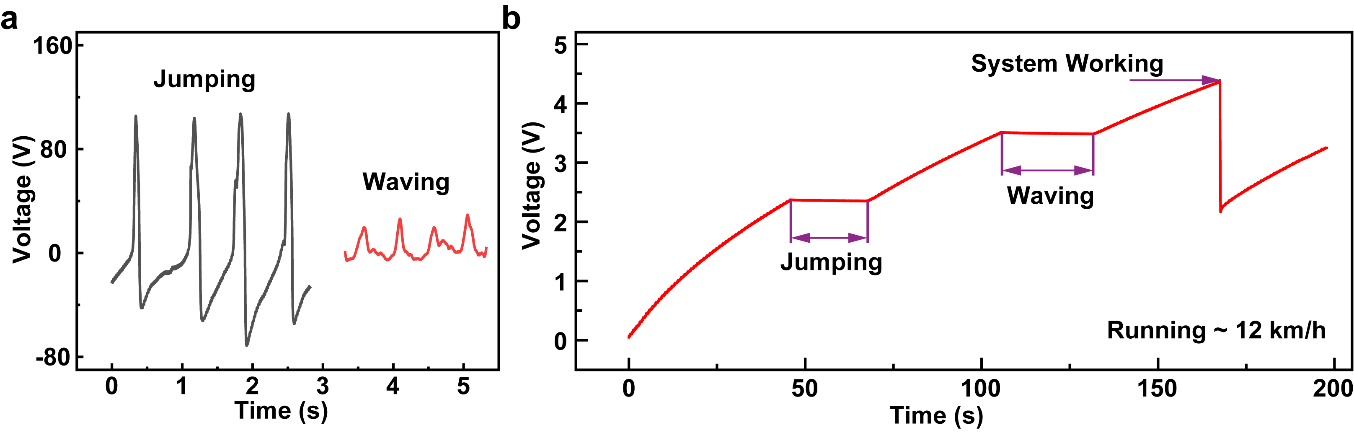


### Figure S18. Effect of different human motions on energy harvesting and system working. a) Voltage output of the *p*TENG during jumping and arm waving. b) Stable energy harvesting from intermittent human motions ensures reliable system working.


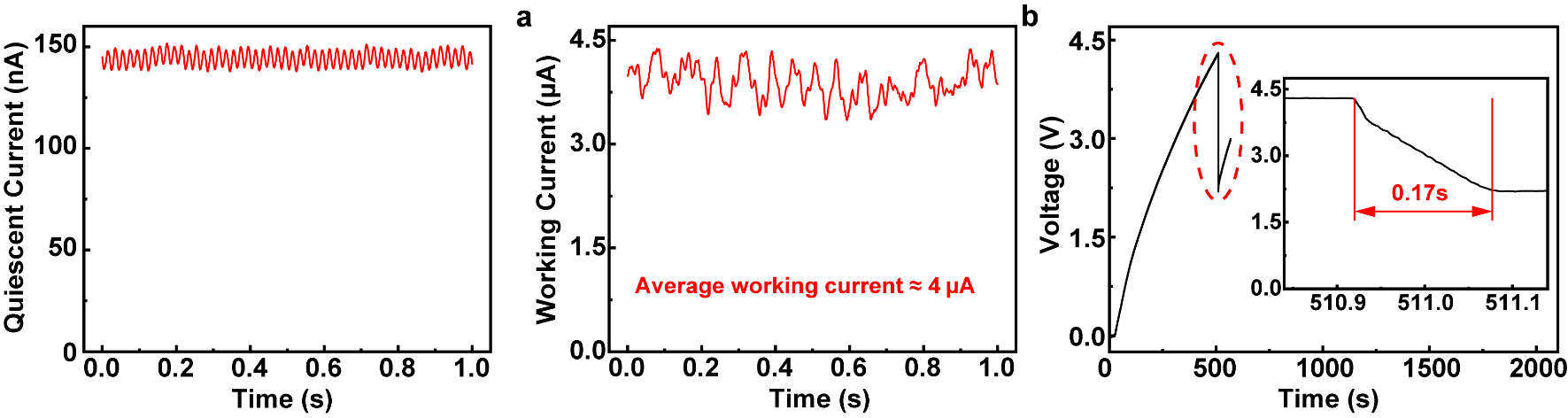


### Figure S**19.** Working current and duration of S_2_ upon activation. a) Working current of S_2_ at 4.4 V. b) Activation time of S_2_.

### Table S1. The performance of different TENGs^[1-21]^.

| Materials | Open Voltage (V) | Area (cm^2^) | Normalized Voltage (V/cm^2^) | Air Permeability (mm/s) | Moisture Permeability (g/m²/day) | Ref. |
| --- | --- | --- | --- | --- | --- | --- |
| PET ^a)^ yarn/ PTFE ^b)^ | 250 | 100 | 2.5 | - | - | 1 |
| Wool fabric/ Silicone | 106 | 20.25 | 5.23 | - | - | 2 |
| MXene/ TPU ^c)^ | 20.1 | 72 | 0.28 | - | - | 3 |
| Nylon/ Polyester | 20 | - | - | - | - | 4 |
| PDMS ^d)^/ Skin | 140.4 | 35 | 4 | - | - | 5 |
| Polypyrrole / Ag | 0.3 | - | - | - | 399.7 | 6 |
| PTFE/ Nylon 66 | 200 | - | - | - | - | 7 |
| SIS ^e)^-SiO_2_ / Skin | 40 | 4 | 10 | - | 300 | 8 |
| PVDF ^f)^/ Nylon 6 | 1000 | 100 | 10 | - | - | 9 |
| PVA ^g)^-HBP ^h)^ / Skin | 52 | 5 | 10.4 | 18 | - | 10 |
| PVDF/ Skin | 113 | 25 | 4.5 | - | 10260 | 11 |
| PVDF-HFP ^i)^/ POM ^j)^ | 255 | 25 | 10.2 | - | - | 12 |
| PVDF-HFP/ Al | 65 | 9 | 7.2 | - | - | 13 |
| PVDF/ Polyamides 6 | 206 | 9 | 22.9 | - | - | 14 |
| PVDF/ PBU ^k)^ | 113 | 25 | 4.5 | - | - | 15 |
| PVDF-HFP/ Al | 400 | - | - | - | 8000 | 16 |
| PAN ^l)^/ LM | 635 | - | - | - | - | 17 |
| PVDF-HFP/ LM | 5.11 | - | - | - | - | 18 |
| PDMS ^m)^ / LM | 91.03 | - | - | - | - | 19 |
| Ecoflex/ LM-PVA hydrogel | 250 | - | - | - | - | 20 |
| Ecoflex/ LM | 35 | - | - | - | - | 21 |
| LMPT/ Nickel | 220 | 6.25 | 35 | 24.5 | 780 | This work |
| ^a)^ Polyethylene terephthalate (PET), ^b)^ Polytetrafluoroethylene (PTFE), ^c)^ Thermoplastic polyurethane (TPU), ^d)^ Polydimethylsiloxane (PDMS), ^e)^ Styrene-isoprene (SIS), ^f)^ Polyvinylidene fluoride (PVDF), ^g)^ Polyvinyl alcohol (PVA), ^h)^ Amino-terminated hyperbranched polymers (HBP), ^i)^ Fluoride-co-hexafluoropropylene (PVDF-HFP), ^j)^ Polyoxymethylene (POM), ^k)^ Polybutadiene-based urethane (PBU), ^l)^ Polyacrylonitrile (PAN), ^m)^ polydimethylsiloxane (PDMS) | | | | | | |

### Table S2. Performance comparison of various electronic switches^[22-26]^.

| Energy conversion method | Voltage and Charging time for capacitor | Active voltage regulation | Application | Ref. |
| --- | --- | --- | --- | --- |
| Rectifier | 10 μF@3.25 V (250 s) | No | Powering a wristwatch | 22 |
| Rectifier | 10 μF@2 V (45 s) | Manual adjustment | Powering low-power electronic timer | 23 |
| Rectifier | 4.7 μF@0.2 V (45 s) | No | Powering LED | 24 |
| Rectifier | 22 μF@2 V (250 s) | Manual adjustment | Powering LCD | 25 |
| Rectifier | 1 μF@17 V (50 s) | Manual adjustment | Powering a wristwatch | 26 |
| SCR & Zener diode | 680 μF@4.4 V (140 s) | Active voltage regulation | Powering a wireless sensing system | This work |

### Table S3. Summarized component parameters of the energy management module.

| **Component** | **Type** |
| --- | --- |
| Rectifier bridge | DB107S |
| Capacitor (C) | 2.2 nF |
| Semiconductor control rectifier (SCR) | EC103M1(Littelfuse) |
| Zener Diode (D) | IN5388(onsemi) |
| Zener Diode (D_1_) | B5817W(CJ) |
| Inductance (L) | 2.2 mH |
| Capacitor (C_1_) | 680 μF |
| Capacitor (C_2_) | 100 nF |
| Triode (Tr_1_) | DTC143ECA(CJ) |
| Zener Diode (D_3_) | B5817W(CJ) |
| Capacitor (C_3_) | 100 pF |
| Resistor (R_1_) | 47 MΩ |
| Monitor chip (Q_1_ and Q_2_) | NTGD1100LT1G(onsemi) |
| Resistor (R_2_) | 10 MΩ |
| Capacitor (C_4_) | 10 nF |
| Capacitor (C_5_) | 100 pF |
| Monitor chip (Q_3_)  Monitor chip (Q_4_) | TPS3839K50DBZR  TPS3839L30DBZR |
| Capacitor (C_6_) | 100 nF |
| Voltage stabilizer (D_2_) | ME6206A25(MICRONE) |

### Table S4. Summarized component parameters of the wireless sensing integrated system.

| **Component** | **Type** |
| --- | --- |
| Temperature sensor | BMP180(Bosch) |
| Chip | STM32L031F6P6(ST) |
| Wireless transmitter | H34S |

### Table S5. Summarized component parameters of the relay terminal module.

| **Component** | **Type** |
| --- | --- |
| Wireless receiver | WR01 |
| Chip | STM32L031F6P6(ST) |
| Wi-Fi Module | ESP01-S |
| Voltage stabilizer | ME6211C33M5G-N |
| Lithium-ion battery | 400 mAh / 3.7 V |

### Reference

[1] C. Chen, L. Zhang, W. Ding, L. Chen, J. Liu, Z. Du,W. Yu, Woven Fabric Triboelectric Nanogenerator for Biomotion Energy Harvesting and as Self-Powered Gait-Recognizing Socks, Energies **2020**, 13, 4119.

[2] L. Dong, M. Wang, J. Wu, C. Zhu, J. Shi,H. Morikawa, Deformable Textile-Structured Triboelectric Nanogenerator Knitted with Multifunctional Sensing Fibers for Biomechanical Energy Harvesting, Adv. Fiber Mater. **2022**, 4, 1486-1499.

[3] Y. Hao, Y. Zhang, A. Mensah, S. Liao, P. Lv,Q. Wei, Scalable, ultra-high stretchable and conductive fiber triboelectric nanogenerator for biomechanical sensing, Nano Energy **2023**, 109.

[4] V. U. Somkuwar,B. Kumar, Influence of the Fabric Topology on the Performance of a Textile-Based Triboelectric Nanogenerator for Self-Powered Monitoring, ACS Appl. Polym. Mater. **2023**, 5, 2323-2335.

[5] C.-R. Yang, C.-T. Ko, S.-F. Chang,M.-J. Huang, Study on fabric-based triboelectric nanogenerator using graphene oxide/porous PDMS as a compound friction layer, Nano Energy **2022**, 92, 106791.

[6] Z. Yu, Z. Zhu, Y. Wang, J. Wang, Y. Zhao, J. Zhang, Y. Qin, Q. Jiang,H. He, Wearable cotton fabric-based single-electrode-mode triboelectric nanogenerator for self‑powered human motion monitoring, Cellulose **2023**, 30, 5355-5371.

[7] M. Zamani, A. Valipouri, S. A. H. Ravandi,A. Alsikh, High‐Performance Double‐Layer Textile‐Based Triboelectric Nanogenerator, Energy Technol. **2024**, 12.

[8] Y. Li, S. Xiao, X. Zhang, P. Jia, S. Tian, C. Pan, F. Zeng, D. Chen, Y. Chen, J. Tang,J. Xiong, Silk inspired in-situ interlocked superelastic microfibers for permeable stretchable triboelectric nanogenerator, Nano Energy **2022**, 98, 107347.

[9] H.-J. Qiu, W.-Z. Song, X.-X. Wang, J. Zhang, Z. Fan, M. Yu, S. Ramakrishna,Y.-Z. Long, A calibration-free self-powered sensor for vital sign monitoring and finger tap communication based on wearable triboelectric nanogenerator, Nano Energy **2019**, 58, 536-542.

[10] P. Jia, L. Wang, X. Yao, D. Zhang, H. Lin, Y. Li, Y. Chen,J. Xiong, Amino-Terminated Hyperbranched Polymer-Based Recyclable Elastic Fibers for a Breathable and Antibacterial Triboelectric Nanogenerator, Macromol. Mater. Eng. **2022**, 307, 2200128.

[11] Z. Li, M. Zhu, J. Shen, Q. Qiu, J. Yu,B. Ding, All-Fiber Structured Electronic Skin with High Elasticity and Breathability, Adv. Funct. Mater. **2020**, 30, 1908411.

[12] Y. Yu, H. Wu, Y. Yu, J. Yan, J. Shi, H. Morikawa, W. Lyu,C. Zhu, Acid- and alkali-resistant, UV-shielding, and photocatalytic self-cleaning nanofiber membrane-based wearable triboelectric nanogenerator for ultra-low-frequency energy-harvesting and self-powered sensors, Chem. Eng. J. **2024**, 490, 151546.

[13] Y. Li, J. Xiong, J. Lv, J. Chen, D. Gao, X. Zhang,P. S. Lee, Mechanically interlocked stretchable nanofibers for multifunctional wearable triboelectric nanogenerator, Nano Energy **2020**, 78, 105358.

[14] S. Chen, Y. Zhang, Y. Li,P. Wang, High-Performance Friction Nanogenerators Based on Electrospun Poly(vinylidene fluoride) Loaded with Microbead Structures, ACS Appl. Polym. Mater. **2024**, 6, 11722-11732.

[15] T. D. Khanh, J. S. Meena, S. B. Choi,J.-W. Kim, Breathable, self-healable, washable and durable all-fibrous triboelectric nanogenerator for wearable electronics, Mater. Today Adv. **2023**, 20, 100427.

[16] F. Jiang, X. Zhou, J. Lv, J. Chen, J. Chen, H. Kongcharoen, Y. Zhang,P. S. Lee, Stretchable, Breathable, and Stable Lead-Free Perovskite/Polymer Nanofiber Composite for Hybrid Triboelectric and Piezoelectric Energy Harvesting, Adv. Mater. **2022**, 34, 2200042.

[17] Q. Ye, Y. Wu, Y. Qi, L. Shi, S. Huang, L. Zhang, M. Li, W. Li, X. Zeng, H. Wo, X. Wang, S. Dong, S. Ramakrishna,J. Luo, Effects of liquid metal particles on performance of triboelectric nanogenerator with electrospun polyacrylonitrile fiber films, Nano Energy **2019**, 61, 381-388.

[18] L. Zheng, M. Zhu, B. Wu, Z. Li, S. Sun,P. Wu, Conductance-stable liquid metal sheath-core microfibers for stretchy smart fabrics and self-powered sensing, Sci. Adv. **2021**, 7, eabg4041.

[19] J. Yang, J. Cao, J. Han, Y. Xiong, L. Luo, X. Dan, Y. Yang, L. Li, J. Sun,Q. Sun, Stretchable multifunctional self-powered systems with Cu-EGaIn liquid metal electrodes, Nano Energy **2022**, 101, 107582.

[20] H.-W. Zhou, C. Zhao, Z.-Y. Zhao, J.-C. Jiang, H.-L. Jin, S. Wang, S. Pan, M.-Y. Xu, Y.-H. Chen,H.-M. Jin, Flexible and multifunctional triboelectric nanogenerator based on liquid metal/polyvinyl alcohol hydrogel for energy harvesting and self-powered wearable human–machine interaction, Rare Metals **2023**, 43, 1186.

[21] Y. Liu, J. Luo, K. Gao, H. Li, P. He,W. Zhao, Triboelectric nanogenerator with liquid metal electrode surface microarray structure for self-powered bidirectional sensing and energy harvester, Mater. Today Commun. **2025**, 44, 112151.

[22] A. R. Mule, B. Dudem, H. Patnam, S. A. Graham,J. S. Yu, Wearable Single-Electrode-Mode Triboelectric Nanogenerator via Conductive Polymer-Coated Textiles for Self-Power Electronics, ACS Sustain. Chem. Eng. **2019**, 7, 16450-16458.

[23] M. Qu, L. Shen, J. Wang, N. Zhang, Y. Pang, Y. Wu, J. Ge, L. Peng, J. Yang,J. He, Superhydrophobic, Humidity-Resistant, and Flexible Triboelectric Nanogenerators for Biomechanical Energy Harvesting and Wearable Self-Powered Sensing, ACS Appl. Nano Mater. **2022**, 5, 9840-9851.

[24] P. Munirathinam,A. Chandrasekhar, Wearable triboelectric nanogenerator for real-time IoT-supported security applications, Sustain. Mater. Technol. **2023**, 37, e00700.

[25] H. Patnam, S. A. Graham, P. Manchi, M. V. Paranjape, Y. S. Huh,J. S. Yu, Highly flexible and harsh temperature-tolerant single-electrode mode triboelectric nanogenerators via biocompatible ionic liquid electrolytes for wearable electronic applications, Adv. Compos. Hybrid Mater. **2024**, 7, 56.

[26] M. U. Khan, S. M. Ansari, H. M. Aldosari,B. Mohammad, 3D copper telluride morphological leaf structure based triboelectric nanogenerator for wearable sensing application, Energy Conv. Manag.-X **2025**, 26, 100976.
